# Supplementary material for: History-dependent switch-like differentiation of keratinocytes in response to skin barrier damage
Source: PLoS Comput Biol. 2025 Jun 9;21(6):e1013162. doi: 10.1371/journal.pcbi.1013162 (PMC12176289; doi:10.1371/journal.pcbi.1013162)
Supplement: S1 Text — (PDF) [file pcbi.1013162.s003.pdf]

SUPPLEMENTARY MATERIAL

History-dependent switch-like differentiation of keratinocytes in response to skin barrier damage

Elisa Domíguez-Hüttinger[1,*], Eliezer Flores-Garza[3], José Luis Caldú-Primo[2], Harley Day[3], Abihail Roque Ramírez[4], and Reiko J Tanaka[3,+]

[1]Departamento de Biología Molecular y Biotecnología, Instituto de Investigaciones Biomédicas, Universidad Nacional Autónoma de México, Ciudad Universitaria, 04510, México, México.

[2]Doctorado en Ciencias Biomédicas, Universidad Nacional Autónoma de México, Ciudad Universitaria, 04510, México, México.

[3]Department of Bioengineering, Imperial College London, South Kensington Campus, London SW7 2AZ

[4]Facultad de Ciencias, Universidad Nacional Autónoma de México, Ciudad Universitaria, 04510, México, México.

Correspondence: * elisa.dominguez@iibiomedicas.unam.mx; + r.tanaka@imperial.ac.uk

Section A1: Construction of the regulatory network for keratinocyte differentiation

We assembled the information from 101 manually curated references into a regulatory network for keratinocyte differentiation (*Figure 1* and *S1 Table*). A key factor driving keratinocyte differentiation is extracellular calcium, which triggers a network of regulatory interactions between PKC, the Epidermal Growth Factor Receptor (EGFR), the AP1 transcription factors (cJun and JunB), p53, Np63, the Notch-Jagged pathway, cMyc, miRNA203 and Stat3. The response of keratinocytes to extracellular calcium can be modulated further by microenvironmental signals such as inflammation and immune responses. Each subsection below describes the details of the information we used to support each arrow of the regulatory network for keratinocyte differentiation.

A.1 Increased extracellular calcium level triggers keratinocyte differentiation

The input for our model is the extracellular calcium level at the basal skin layer, which consist of undifferentiated keratinocytes. Extracellular calcium is sensed by keratinocytes via Calcium-activated Receptor (CaR) that activates Protein-Kinase C (PKC). In some experiments, PKC can also be activated by the pharmacological agonist 12-O-Tetradecanoylphorbol-13-acetate (TPA) (1).

In vivo, extracellular calcium level is increased by skin barrier damage. In a healthy and intact epidermis, extracellular calcium forms a gradient, with the lowest concentrations in the basal layer and the highest at the interface between the granular and corneal layers. When the skin barrier is compromised, the extracellular calcium level rises in all layers of the epidermis, including the basal layer. It occurs partly by

passive mechanisms (increased flux of extracellular calcium caused by breakage of desmosomes in the skin barrier and of tight junctions in the viable layers of the epidermis) (2) but also by active release of intracellular calcium stored in the endoplasmic reticulum (3).

In vitro, the increased calcium level in the basal layer induced by skin barrier damage can be mimicked by the extracellular addition of calcium to the medium for isolated cells or cell lines. In those so-called in vitro calcium-switch experiments, a sustained increase in the extracellular calcium level triggers differentiation of undifferentiated keratinocytes (e.g. primary human keratinocytes) (4). For example, calcium challenges in hNEK cells resulted in a transient increase in the expression levels of skin barrier precursors (involucrin, filaggrin, transglutaminase) peaking at 3 days post calcium challenge in a Stat3-dependent manner (5,6). Analogously, upregulation of genes for lipid metabolism (ACBP, IGFBP1) was observed 24h post-10-minute acetone treatment in tissue-engineered skin equivalents ((7), Fig. 5). In vivo, the skin barrier disruption induced by sodium dodecyl sulphate application increased cornification-related protein expression (8). Along the same line, barrier damage triggers the expression of AMP. For example, the AMP (RNAse 7 and hBD-3) expression increased 24h post barrier disruption in vivo ((9), Fig. 4A) but not if the barrier was occluded. HBD3 expression increased in human keratinocytes from donors cultured for 5-7 days under high calcium (1.3 mmol/L calcium) ((10), Fig. 3B) corresponding to the gradual differentiation and upward movement of keratinocytes from the basal towards the granular layer of the epidermis.

A.2 Regulation of EGFR phosphorylation

In healthy epidermis, the Epidermal Growth Factor Receptor (EGFR) is activated by phosphorylation in the basal layer. EGFR activity contributes to maintaining keratinocytes being undifferentiated. EGFR overactivation (and the ensued under-differentiated epidermis), which is commonly observed in carcinomas (e.g. Squamous Cell Carcinomas), occurs possibly due to mutations that affect either EGFR copy number or EGFR tyrosine kinase domain. Mutations in the EGFR tyrosine kinase domain lead to constitutive activation of EGFR (i.e. signalling without the need of a ligand) (11). Even in the absence of mutations, alterations in the tumour microenvironment by UV light or by enrichment of growth factors can further overactivate EGFR. The clinical relevance of EGFR-mediated under-differentiation of keratinocytes is further evidenced by the fact that there are therapeutic agents for cancer treatment that target EGFR (11).

EGFR phosphorylation (activation) is:

- inhibited by PKC as shown in (12) where the EGFR phosphorylation level peaks at 2-15 minutes post-stimulation with PKC inhibitor (GF 109203X) and with the EGFR ligand angiotensin in IEC18 cells (rat-derived ileum-epithelial cell line).

- inhibited by p53. The EGFR expression level increased when more p53 inhibitor was added (13).
- induced by EGFR itself. This positive feedback is mediated by amphiregulin (AREG), which is produced in response to EGFR activation and induces EGFR signalling (14).

In addition to the processes described in Fig 1, EGFR phosphorylation is

- induced by growth factors secreted and pre-processed through activating cleavage by matrix metalloproteinases in the extracellular matrix mainly in the dermis (basal lamina) (14).

A.3 Regulation of p53 transcription

p53 transcription is:

- inhibited by c-Jun as demonstrated in primary human keratinocytes (15)
- inhibited by Np63 (particularly the ΔNp63 isoform) by competitive inhibition (16)
- inhibited by Notch (17).
  - In vivo measurements on a mouse model of leukaemia showed a dramatic increase in p53 protein level from 24 hours and peaked at 36 hours after initiation of doxycycline (a suppressor of Notch transgene) ((18), Fig. 4).
  - Notch-mTOR-PI3K-Akt/PKB-p53 decreases the activity of p53 (i.e. its binding ability to DNA) by post-translational modifications (phosphorylation of specific sites) of p53 ((19,20), Fig. 2).
  - repressed by Notch via induction of its transcriptional repressor, RBPJk (21).
- induced by miRNA203 via Snai1, which is inhibited by miRNA203 (22) and inhibits p53 by binding to its DNA binding site (23).

A.4 Regulation of the Notch-Jagged pathway

The Notch-Jagged pathway is important in regulating the stemness of epithelial tissues (24). In the epidermis, the Notch-Jagged pathway controls cell proliferation, growth arrest and early differentiation. Loss of the Notch pathway activity (for example, by Notch *ko*) leads to hyperplasia (manifested as increased epidermal thickness) and aberrant keratinocyte differentiation (increased expression of terminal differentiation markers, filaggrin and involucrin, and reduced expression of the early differentiation marker, keratin 1 (25–27)). The critical role of Notch on keratinocyte differentiation is shown in (28), where keratinocyte-specific deletion of ADAM17 (MMP that activates Notch by transactivation, i.e. in a delta-ligand-independent manner) in mice leads to the spontaneous development of AD symptoms.

Notch is:

- activated by PKC in the epidermis (25,29). This activation has been observed also in chicken neurons, where PKC favours the nuclear translocation of Notch previously stimulated with Jagged (30) and

during the establishment of left-right polarity in chicken gastrulation (31). It has also been observed that, in murine chondroprogenitor cells, the canonical Notch-responsive Hes1 transcription factor is transcriptionally induced by the Calcium-calmodulin pathway, in which PKC is involved (32).

- induced by p53, as shown in (27), where transcriptional control of Notch by p53 is supported by strong experimental evidence, including a detailed analysis with Chip experiments and expression assay when p53 is silenced.
- induced post-translationally by its canonical ligand, Jagged (33).
- Induced post-transcriptionally by cMyc (34).

In addition to the processes described in Fig 1, Notch is

- modulated by the stiffness of the extracellular matrix. Soft extracellular matrix or high confluence inhibit YAP/TAZ activity, which inhibits Hes1 expression, where Hes1 is a key transcriptional inductor for Notch (35).

The ligand Jagged (as well as the ligand Delta) binds and activates Notch. Notch is then cleaved extracellularly by metalloproteases, such as ADAM, and intracellularly by gamma-secretase, to separate the notch intracellular domain (NICD). NICD then translocates to the nucleus, activating the transcription factor, CSL, by removing the co-repressor, thereby inducing the expression of CSL's target genes, such as the transcription factors Hes and Hey (36).

The levels of expression of Jagged are:

- induced by ΔNp63 by transcriptional regulation. A time course of Jagged1 [0,3,6,9,12,24,36]h post adenovirus-mediated transfection of A172 human glioma cells with ΔNp63 ((37) Fig. 2, left) demonstrates a steady increase of Jagged1, with the highest level at 36h post-transfection. Overexpression of Jagged in the presence of p63 (transfected with adenovirus) was also observed in the seven human cancer cell lines, DLD1, colo320, HCT116 (colorectal cancers), Saos2 (osteogenic sarcoma), H1299 (lung cancer), PLC/PRF5 (hepatocellular carcinoma) and A172 (glioma). Chromatin immunoprecipitation confirmed the existence of binding site for p63 in the second intron of the Jagged1 promoter. Analogously, microarray data shows that p63 overexpression in the SAOS2 cell line induces a 3-fold increase in Jagged1 levels hours after transfection with TAp63. These microarray results were confirmed by RT-PCR analysis (38).
- induced by p53. Transcriptional regulation was confirmed by time-course data in MCF7 breast cancer cell lines where p53 induces Jagged expression within 6-36 hours post-stimulation (39).

A.5 Regulation of cMyc expression

Myc is a protooncogene. Its amplification has been found in 50% of Squamous Cell Carcinomas (40). cMyc induces proliferation of stem cells, as is consistent with the gene expression signatures associated with overactivation of cMyc observed in microarray experiments: The majority of genes that cMyc upregulates are involved in cell growth and proliferation; Most of the genes down-regulated by cMyc are cell adhesion markers (i.e. cMyc might promote differentiation by the inhibition of adhesion molecules in the basal layers) (41).

The expression of cMyc is:

- induced by Notch (42).
- activated by ΔNp63 (43).
- activated by beta-catenin/APC (44).

An in vitro calcium challenge experiment with Normal Human Epidermal Keratinocytes (NHEK) (6) shows that cMyc peaks on day 1 and decays quickly, suggesting that cMyc is expressed early during the keratinocyte differentiation process. In vivo, cMyc expression is restricted to the basal or spinal layer (45).

A.6 Regulation of Np63 expression

The p63 gene has two promoters, generating two protein isoforms, TAp63 and ΔNp63. Relevant for epidermal differentiation is ΔNp63, where Δ denotes that this isoform (Np63) is truncated, lacking the transactivation domain (TA), but has the same DNA and protein binding sequences as the active isoform p63. ΔNp63 acts as a repressor of p53 (which also has the same DNA and protein binding sequences as the active isoform p63) and of Np63 activity by competitive inhibition (i.e. ΔNp63 can occupy the DNA and protein binding sites for p53 and p63, but without transducing the signal due to the lack of the transactivation domain). ΔNp63 plays a key role in epidermal development since the complete abolishment of p63 is a lethal mutation. Mice bearing this mutation cannot survive due to severe dehydration, which is attributed to a deficient skin barrier (16). p63 is located in a chromosomic region amplified in various cancers (46).

The expression of Np63 is:

- inhibited by miRNA203 (Fig. 4b in (47)). A calcium-mediated decrease in p63 expression is almost restored to its basal (low) calcium levels when miRNA203 is blocked under high calcium conditions. The normalized expression level of p63 is 1 : 0.3 : 0.8 for low calcium : high calcium : high calcium + miR203. Primary mouse keratinocytes show a miR203 increase and ΔNp63 decrease in expression

for days after being challenged with 1.2 mM CaCl (Fig. 1 in (48)) and miR203 targets p63 mRNA at 3'UTR (Fig. 2 in (48)). This miRNA203-mediated inhibition of p63 expression under high calcium conditions was also observed in NHEK (49).

- induced by Stat3, which in turn transcriptionally activates Np63 (positive feedback loop) (50).
- induced by cMyc through the transcriptional activation of the histone-methyltransferase, Setd8. Methylation of the target promoter of Np63 by Setd8 activates the expression of Np63 (51) via the p300 protein (52,53). p300 also mediates the transcriptional activation of NFkB-dependent Protease Activated Receptor (PAR2) genes, including the pro-inflammatory cytokine IL8 via p300-mediated NFkB activity (54).
- inhibited by Notch (Fig. 5G right in (55), (56), Fig 6B right in (57)).
- induced by EGFR. EGFR induces cMyc via activation of ΔNp63 (58).

In addition to processes described in Fig 1, the expression of Np63 is

- induced by Keratinocyte Growth Factor (KGF) in a p38-dependent manner, leading to increased proliferation (59). Ex vivo limbal cells (stem cells that give rise to corneal epithelial cells) show an increase in proliferation and in ΔNp63 expression 24h post-stimulation with different doses (0, 1, 5, 10, 20 ng/ml) of KGF (Fig. 6a in (59)). KGF is a member of the Fibroblast Growth Factor family, secreted by fibroblasts in the dermis and acts on epithelial cells as a potent mitogen (i.e. stimulating proliferation). KGF expression is augmented in response to wounding; its secretion is induced by the pro-inflammatory cytokine IL1 via induction by cJun and inhibition by junB (60). Epidermis-specific knockout of KGFR shows a high atrophy and disorganized epidermis, with reduced keratinocyte proliferation. This interplay between fibroblast growth factors and keratinocytes plays an important role in the context of wound healing (61).

A.7 Regulation of cJun and junB expression

The transcription factors (TFs), cJun and junB, belong to the AP1 family and play a pivotal role in keratinocyte differentiation. Deletion of either of them induces psoriasis-like lesions (62). These AP1 factors can induce epigenetic changes that regulate the accessibility of TFs to their target sequences in basal vs. differentiated cells ((63), Fig. 3) where p63 and AP1 binding sites are present in active (i.e. accessible) enhancers of differentiated cells only. In vivo, the expression of cJun is restricted to the granular layer (64).

c-Jun expression is:

- induced by EGFR. c-Jun is the canonical downstream TF of EGFR. For example, EGFR inhibits its target gene, Notch1, via the activation of c-Jun, which represses p53 (i.e. EGFR induces cJun which inhibits p53 that induces Notch) (15).
- induced by PKC ((65), Fig. 3b right).
- repressed by the action of its transcriptional antagonist, JunB (66).

In addition to the processes described in Fig. 1, c-Jun expression is

- induced by interleukin 1 (IL1) in a post-transcriptional way via JNK (IL1 activates JNK, which phosphorylates cJun and thus increases its activity) (67) and also via induction of transcription of cJun (68).
- induced by TNFα (63); this process is responsible for the TNF-mediated inhibition of terminal differentiation markers (69).

JunB expression is:

- induced transcriptionally by PKC (66), demonstrating its involvement in the calcium-activated PKC keratinocyte differentiation. This transcriptional induction of JunB expression by PKC is also shown in (70), using TPA, the pharmacological agonist for PKC.

A.8 Regulation of miRNA203 expression

miRNA203 is a skin-specific miRNA overexpressed in psoriasis but not in atopic dermatitis (65). It also plays a crucial role in metastasis since its levels are downregulated in tumour samples and metastatic prostate cancer cell lines, inhibiting migration and invasiveness (71). Molecularly, this effect can likely be attributed, at least in part, to the inhibition of the mesenchymal marker Snai2 by miRNA203 (72). miRNA203 also plays a key role in murine skin in response to wounding (73).

miR203 is not expressed in proliferating epidermis and restricts keratinocyte proliferation (74). Furthermore, Human Papilloma Virus (HPV) infection decreases the expression of miR203 in Normal Human Keratinocytes (NHKs) (49), possibly via the interference of E6 viral protein with p53 expression, partly explaining the mechanisms by which HPV impairs stratified epithelium function (75).

miRNA203 expression is:

- induced transcriptionally by JunB, which acts as a transcriptional activator of miRNA2013 ((70), Figs. 2g and 3).

- induced transcriptionally by p53. miR203 expression is reduced both in the absence of p53 (iRNA) and also when the HPV viral protein E6 (which inhibits p53 activity) is added (75). This partly explains why miR203 is diminished in stratified epithelia infected with HPV (49).
- induced transcriptionally by p63 via the induction of expression DICER (76), an inducer of expression of miR-203 (77).

A.9 Regulation of Stat3 activation

Stat3 is a key modulator of keratinocyte differentiation (78).

- Stat3 activity is induced by ΔNp63 by phosphorylation (50).
- Stat3 expression is induced by miR203 via the downregulation of the Stat3 repressor SOCS3 (48,65,79–83).

A.10 Regulation of Terminal Differentiation Markers

Keratinocyte differentiation culminates in the expression of terminal differentiation markers, including skin barrier components such as filaggrin, loricin, corneodesmosomes and antimicrobial peptides such as RNASE7, S100A7 and SLPI. Regulation of the terminal differentiation markers occurs in a coordinated, synchronous, and spatially overlapping manner in the stratified epidermis (84–89) as they form part of the terminal differentiation complex. In a healthy epidermis, the terminal differentiation markers are expressed in the uppermost keratinocyte layer, termed the granular layer. In our model, we group all the terminal differentiation markers into a single variable, TDM (for Terminal Differentiation Markers), to reflect their common dynamics and regulation. The outputs of the regulatory network proposed in this manuscript are TDM. Among them, we specifically focus on pro-Filaggrin and AMP, as they are skin barrier precursors which play a key role in epidermal homeostasis.

TDMs are:

- inhibited transcriptionally by Stat3, which directly binds to the promoter and decreases the expression of Flg (90).
- induced epigenetically by p63. p63 directly induces the expression of Satb1, which regulates the chromatin architecture of the epidermal differentiation complex by direct binding (91).

A.11 Regulation of markers of the epidermal basal layer

Keratins 5 and 14 (K5K14) are markers of the basal layer. Their expression is:

- induced by ΔNp63 (92). p63 directly induces expression of K5K14 by binding to an enhancer in the 5' upstream region.

A.12 Regulation of markers of the epidermal spinal layer

Keratins 1 and 10 (K1K10) are markers of the spinal layer. Their expression is incoherently regulated by calcium signalling:

- induced by calcium via Notch in a Hes1-dependent way (29)
- inhibited by TPA-mediated PKC activation (65).

A.13 Effects of innate immune responses on keratinocyte differentiation

Innate immune responses (NFkB stimulated by pathogens) induce the expression of terminal differentiation markers. NFkB has a dual regulation of Np63 at the transcriptional level: It acts as a transcriptional repressor by directly binding to the Np63 promoter (57) and by inducing the proteasome-mediated degradation of Np63 (93). It also acts as a transcriptional activator by inducing the acetylation of the Np63 promoter through the activation of p300 (52,94,95). We assume that the net effect is positive because epigenetic modifications such as acetylation are more persistent.

Innate immune responses result in an increased production of terminal differentiation markers. In vitro, TLR3 activation of NHEK cells sharply increases the expression of barrier-related genes, specifically those related to lipid metabolism (e.g., UGCG, SPTLC, and ACACA), 24h post-stimulation ((96), Fig. 3), and challenge with *S. epidermidis, S. aureus, and P. acnes* on a reconstructed human epidermal model, by either applying the bacteria on the model surface (intact barrier) or adding them to the culture medium (simulating barrier breach), induced dose-dependent changes in the expression of terminal differentiation makers 24h post-pathogen-challenge (Fig. 3 in (97)). *P. acnes* triggers expression of the anti-microbial peptides (AMP) human beta defensin (hBD)-2, LL-37 via the activation of PAR2 in the granular layer of the epidermis (98). *Ex vivo,* the mRNA level of the AMP (CCL20 and hBD2) increased in response to *P. gingivalis* for 16 hours in a PAR2-dependent way. The experiments were performed in a primary cell culture of gingival epithelial cells isolated from healthy patients and cultivated with a low (0.15mM) calcium concentration (Figs. 4,5 in (99)). In vivo, immortalized keratinocytes (HaCaT cells) treated with *P. acnes* for 12 hours showed PAR2-dependent increasing in mRNA levels of hBD-2 and LL-37, with a peak expression after 12 hours ((98), Fig. 5). S100A8 and S100A9 mRNA expression was also upregulated by 16h of TLR5 ligand stimulation with purified flagellin of human primary keratinocytes cultured either in monolayer or in organotypic cell cultures; increased expression was observed regardless of the keratinocyte differentiation status but prevented by TLR5 knockdown (Figs. 4,6 in (100)). The mRNA of the AMP beta-defensin shows a sustained increase at 8, 16 and 24h upon stimulation with *S. aureus* and *S. epidermis*, in human primary keratinocytes where differentiation was induced by calcium, in a TLR2 dependent way ((101), Fig. 1).

Section B: Boolean model of keratinocyte differentiation

The Boolean model of keratinocyte differentiation (*Figure 1B*) is given by the discrete dynamical system,

$$\boldsymbol{x(t+1)} = \boldsymbol{F}\big(\boldsymbol{x(t)}\big),$$

with $\boldsymbol{x(t)} \in \{\boldsymbol{0},\boldsymbol{1}\}^{\boldsymbol{8}}$ representing the vector of the discrete state variables $[EGFR, cJun, p53, miRNA203, Notch, cMyc, Np63, Stat3](t)$ governed by the discrete dynamical system:

Supplementary Equation 1: *Boolean model of keratinocyte differentiation*

```
EGFR(t+1)=  !PKC(t) | !p53(t) | EGFR(t)
cJun(t+1)= PKC(t) | (!PKC(t) & !p53(t) & EGFR(t)) & cJun(t)
p53(t+1)= PKC(t) & (!cJun(t) | !Np63(t) | !Notch(t) | miRNA203(t))
miRNA203(t+1)=  (Np63(t) | p53(t)) &  !cJun(t)
Notch(t+1)= (p53(t) | Np63(t)) & cMyc(t)
cMyc(t+1)= Notch(t) | Np63(t)
Np63(t+1)= cMyc(t) & (!Notch(t) | EGFR(t))  & (!miRNA203(t) | Stat3(t) ) & !NFkB(t))
Stat3(t+1)= miRNA203(t) & (cMyc(t) & (!Notch(t) | EGFR(t))  & (!miRNA203(t) |
Stat3(t) )     & !NFkB(t)))
FLG_AMP(t+1)=  !Stat3(t) & Np63(t)
```

where `!`, `&` and `|` represent the *not*, *and* and *or* Boolean operators, respectively. The input and output of the model are the vector [`PKC,NFkB`] (t) and the dynamical variable (`FLG_AMP(t)`), respectively.

Most processes described in the model correspond to transcriptional regulations and are assumed to occur at the same discrete time scale ($t = 1,2,\ldots,N$). The transcriptional regulation of y by x is described by $y(t+1)$ being a function of $x(t)$. Other processes are phosphorylations for Stat3 and cJun (or JunB) and methylations for Np63 that occur significantly faster and slower, respectively, than transcription. To represent the faster process of phosphorylation of y activated by x, we assume that $y(t+1)$ is a function of $x(t+1)$. To model the slower process of methylations of $Np63$ by x, we initially assumed time-scale separation due to epigenetic modification: $Np63(t+1)$ is a function of an additional variable for an open chromatin state, $pre\_Np63(t)$ and that $pre\_Np63(t+1)$ is a function of $x(t)$. However, we did not include this additional variable in the model (Supplementary Equation *1*) since its inclusion did not alter the results.

The network has 9 state variables but the Boolean model has only 8 state variables, except for JunB. It is because JunB has only one regulator, cJun, which inhibits JunB. We therefore described the effects of JunB as !cJun instead of including JunB as a state variable.

Section C: Analysis of the Boolean model of keratinocyte differentiation

Dynamic simulations of the Boolean model (Supplementary Equation 1) were conducted in `Rstudio` (ver. 1.4.1717) using the `R` package `BoolNet` ver. 2.1.5 (102). Attractors and the size of their basin of attraction were found by exhaustively reconstructing the synchronous dynamics of each of the $2^{n=9}$ states until an attractor is reached and assigning each initial condition to its resulting attractor. We confirmed that the fixed-point attractors are maintained under an asynchronous update regime.

Section D: Network reduction to derive the keratinocyte differentiation motif

We extracted the key structure of the regulatory network by the kernel reduction methodology (103), i.e. sequential substitution of nodes by its regulatory interactions through identification of local inputs and outputs to those nodes and absorbing nodes with in-degree >1 and out-degree >1 into regulatory interactions (connecting the local inputs to the local outputs). The essential regulatory structure was obtained by 10 steps described below. We confirmed the convergence to the same minimal structure irrespective of the order of the steps.

We start with the 9-dimensional network represented in *Figure 1B*. Their regulatory interactions can be represented by a 9x9 regulatory matrix below, where +/- of the (*i, j*)-th element represent activation and inhibition of the *i*-th node to the *j*-th node, respectively.

	EGFR	p53	Notch	cMyc	**Np63**	**Stat3**	miR203	JunB	cJun
EGFR	+				+				+
p53	-		+				+		
Notch		-		+	-				
cMyc			+		+				
**Np63**		-	+	+		+	+		
**Stat3**					+				
miR203		+			-	+			
JunB							+		-
cJun		-						-	

Step 1: Absorbing the miR203 node

We start the reduction process by absorbing the miR203 node because we do not have data describing its dynamics. miR203 is positively regulated by its 3 local inputs (JunB, Np63 and p53) and regulates its 3 local outputs (Stat3, Np63 and p53).

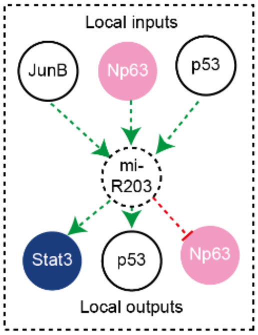

The miR203 node can be absorbed by substituting the miR203 node by 9 (3 inputs × 3 outputs) regulatory interactions via miR203, that are summarised in the table below with bold + and – representing the activation and inhibition, respectively, and shown by dotted arrows in the figure below. Regulatory interactions that were present already in the original network are shown in non-bold font in the table below.

		Local outputs for miR203		
		**Stat3**	**Np63**	**p53**
Local inputs for miR203	**JunB**	+	-	+
	**Np63**	++	-	-+
	**p53**	+	-	+

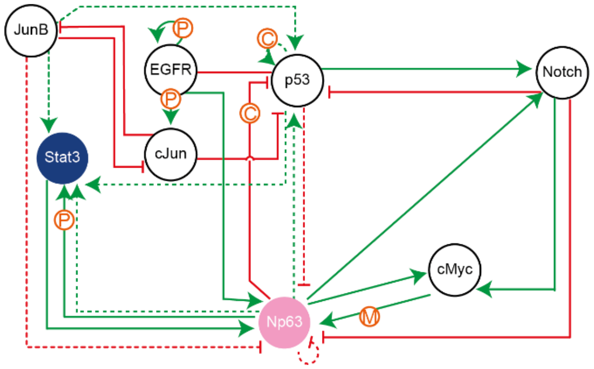

The size of the regulatory matrix is now reduced to 8x8 as we removed the miR203 node. The newly added 9 positive and negative regulatory interactions are shown in bold red and green, respectively, on a highlighted black background. The net regulation of p53 by Np63 is incoherent (shown in the orange background) because the new regulation of p53 by Np63 is positive while it was negative the original network.

	EGFR	p53	Notch	cMyc	Np63	Stat3	JunB	cJun
EGFR	+				+			+
p53	-	**+**	+		**-**	**+**		
Notch		-		+	-			
cMyc			+		+			
Np63		-**+**	+	+	**-**	+**+**		
Stat3					+			
JunB		**+**			**-**	**+**		-
cJun		-					-	

Step 2: Absorbing the cJun node

The cJun node has two inputs (JunB and EGFR) and two outputs (JunB and p53). It is hence substituted by four regulatory interactions via cJun. The regulation of JunB by JunB itself is positive due to double inhibition (JunB inhibits cJun, which inhibits JunB). Similarly, the regulation of p53 by JunB is also by double inhibition (JunB inhibits cJun, which inhibits p53). In the table below, the activation of p53 by JunB is described as +**+** because the corresponding arrow was already identified in Step 2 above and appears for the second time (and shown in non-bold).

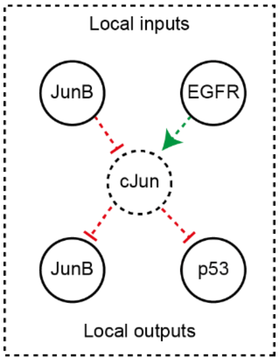

		Local outputs for cJun	
		**JunB**	**p53**
Local inputs for cJun	**JunB**	**+** (double inhibition)	+**+** (double inhibition)
	**EGFR**	**-**	**-**

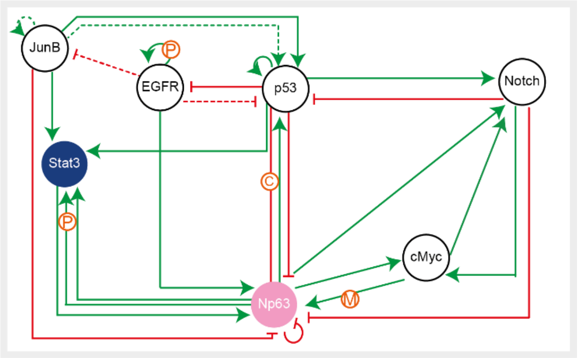

The size of the regulatory matrix is now reduced to 7x7 as we removed the cJun node. The newly added 4 positive and negative regulatory interactions are shown in bold green and red font, respectively, on a highlighted black background.

	EGFR	p53	Notch	cMyc	Np63	Stat3	JunB
EGFR	+	**-**			+		**-**
p53	-	+	+		-	+	
Notch		-		+	-		
cMyc			+		+		
Np63		-+	+	+	-	++	
Stat3					+		
JunB		+**+**			-	+	**+**

Step 3: Absorbing the Notch node

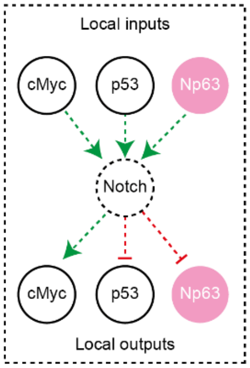

The Notch node has three inputs (cMyc, p53, and Np63) and the same three outputs (cMyc, p53 and Np63). It is hence substituted by 9 regulatory interactions via Notch. Newly identified regulations (positive or negative) that did not appear in the previous steps (including in the original network) are marked as bold "+" or "-". This results in incoherent regulations of Np63 by cMyc and of p53 by Np63 and by p53, in double activations of cMyc by Np63, and in double inhibitions of Np63 by Np63 and by p53.

Local inputs for Notch	Local outputs for Notch:	cMyc	Np63	p53
	**cMyc**	+	+-	-
	**Np63**	++	--	+--
	**p53**	+	--	+-

The resulting 6X6 regulatory network and its corresponding matrix are shown below.

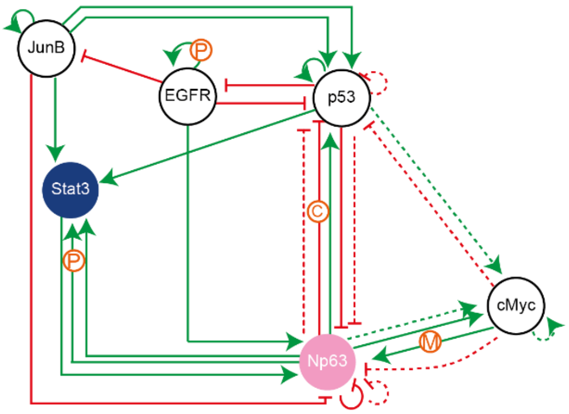

	EGFR	p53	cMyc	Np63	Stat3	JunB
EGFR	+	-		+		-
p53	-	+**-**	**+**	-**-**	+	
cMyc		**-**	**+**	+**-**		
Np63		+-**-**	+**+**	-**-**	++	
Stat3				+		
JunB		++		-	+	+

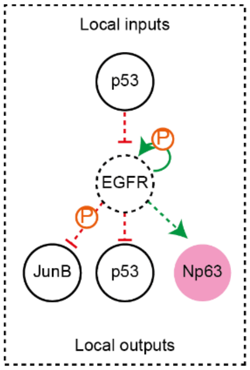

Step 4: Absorbing the EGFR node

The EGFR node has only one input (p53) and three outputs (JunB, p53 and Np63). It is substituted by the following three regulations via EGFR. Newly identified regulations (positive or negative) that did not appear in the previous steps (including in the original network) are marked as bold “+” or “-”.

Local outputs for EGFR

		JunB	p53	Np63
Local input for EGFR	**p53**	+	-++	---

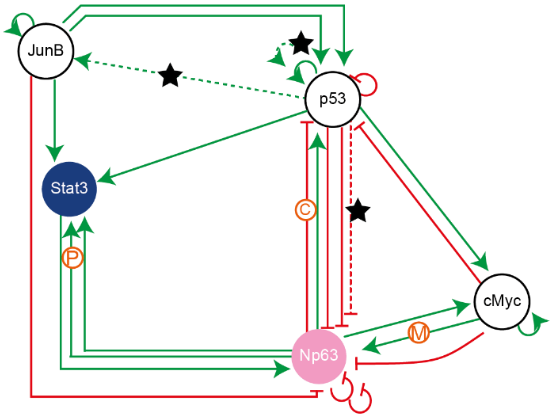

The EGFR node has a positive self-feedback loop, making all EGFR-mediated regulations possible sources of cooperativity, which will play a role in the choice of kinetic terms of the mathematical model (law of mass action vs. hill equations). To keep track of the possible sources of cooperativity, we mark those regulations that include positive self-feedback with a star in the regulatory network.

	p53	cMyc	Np63	Stat3	JunB
p53	-++	+	---	+	+
cMyc	-	+	+-		
Np63	+--	++	--	++	
Stat3			+		
JunB	++		-	+	+

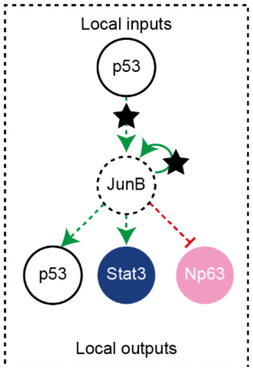

Step 5: Absorbing the JunB node

The JunB node has only one input (p53) and three outputs (p53, Stat3 and Np63). It is substituted by the following three regulations via JunB.

Local outputs for JunB

		p53	Stat3	Np63
Local input for JunB	**p53**	-++**+**	++	---**-**

	p53	cMyc	Np63	Stat3
p53	-+++	+	----	++
cMyc	-	+	+-	
Np63	--+	++	--	++
Stat3			+	

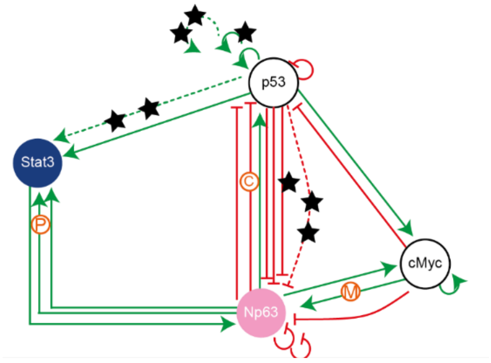

Step 6: Absorbing the cMyc node

The cMyc node has two inputs which are also its outputs (p53 and Np63), and the cMyc node is substituted by the 4 regulatory interactions.

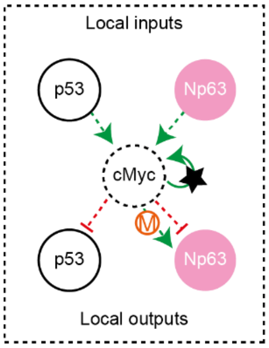

		Local outputs of cMyc	
		**p53**	**Np63**
Local inputs of cMyc	**p53**	--+++	-----+
	**Np63**	---+	---+

Np63 is regulated by cMyc incoherently and through different biochemical mechanisms: the positive regulation is via DNA methylation-dependent epigenetic regulation and the negative regulation is via faster transcriptional repression. The difference in time-scales of the regulations is important in formulating the mathematical model. For example, quasi-steady state approximations can be used for faster reactions, and the integral-over-time serves for representation of slower reactions (104).

At this stage, most of the interactions between the nodes are incoherent. To help us elucidate the net effect, we count the number of positive and negative interactions.

	p53	Np63	Stat3
p53	+++--	-----+	++
Np63	+---	---+	++
Stat3		+	

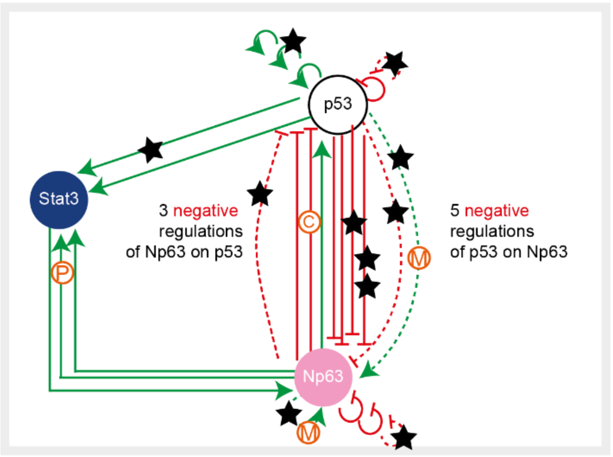

Step 7: Absorbing the p53 node

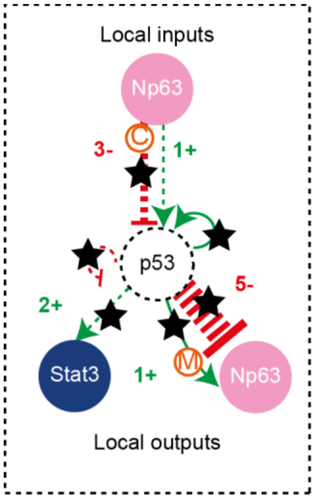

The p53 node has one input (Np63) and two outputs (Stat3 and Np63), and the p53 node is substituted by 6 regulations.

		Local outputs of p53		
		**Stat3 (2+)**	**Np63 (+) weaker**	**Np63 (5-) stronger**
Local inputs of p53	**Np63 (3-), stronger**	6-	3-	15+ (double inhibition)
	**Np63 (+), weaker**	2+	+	5-

We consider two inputs, Np63(3-) and Np63(+), because Np63 regulates p53 both positively and negatively (incoherent regulation), where 3 '-'s represent 3 ways the negative regulation of p53 by Np63 occurs. They are two transcriptional events (via Notch and cMyc) and one competitive inhibition between Np63 and p53. The positive regulation is transcriptional via miR203.

We consider three outputs, Stat3(2+), Np63(+) and Np63(5-), because the output Stat3 is regulated by two independent positive regulations and the output Np63 is incoherently regulated by p53 with the positive regulation through epigenetic activation and the negative regulation through transcriptional repression enacted by 5 intermediate molecules (hence "5-").

This reduction process results in the 2-node network of Stat3 and Np63.

	**Np63**	**Stat3**
**Np63**	**11-/17+**	**6-/4+**
**Stat3**	+	

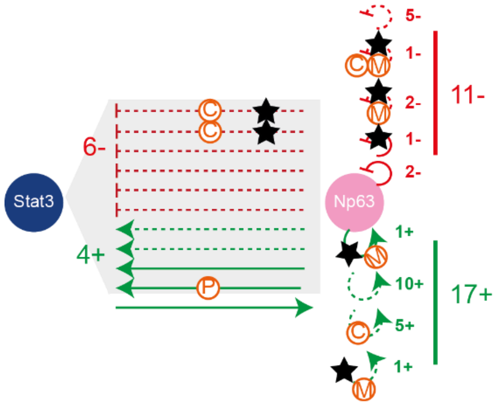

This network has the following regulations:

(1) Stat3 induces Np63,

(2) Np63 induces and also inhibits Stat3, and

(3) Np63 regulates itself through a positive and negative feedback.

For the incoherent regulation (2), we decided to keep only the positive effect of Np63 on Stat3 (Np63 induces Stat3) because the net effect of Np63 on Stat3 has been reported as positive (50). Furthermore, adding the negative effect in a mathematical model did not alter the main features of our interest, including observation of the bistable behaviour and the fit to data (*Figure F in S1 Text*). For another

incoherent regulation (3), we assumed that the net effect of Np63 on itself is positive because the number of positive feedback loops (17) is much larger than that for the negative ones (11).

We completed the reduction process of the state variables and shall move to plugging back the input and the output nodes of the network in Steps 8-10 to derive the *keratinocyte differentiation motif* represented in *Figure 2A*.

Step 8: Plugging back the calcium input (PKC) node

We incorporated the calcium (PKC) node back as an input of the reduced network by explicitly representing its direct regulations on the nodes that are still present in the reduced network (PKC induces Notch, inhibits EGFR and induces JunB). Next, we sequentially incorporated those PKC-mediated direct regulations of Notch, EGFR and JunB into the local outputs of those nodes (cMyc, p53 and Np63 for Notch; JunB, p53 and Np63 for EGFR; and p53, Stat3 and Np63 for JunB). A visual representation of each of these sequential steps can be found in *Figure A*.

The resulting effects of the calcium input (PKC) on the keratinocyte differentiation motif are:

- Calcium input (PKC) induces Stat3.
- Calcium input (PKC) incoherently regulates Np63. It inhibits Np63 by 4 regulatory interactions and activates Np63 by one interaction that is methylation-dependent. In the mathematical model, we assume that the net effect of calcium input on Np63 is inhibitory because the number of negative regulation (4) is much larger than that for the positive ones (1).

Step 9: Plugging back the infection input (NFkB) node

Plugging back the infection input (NFkB) node into the reduced network is trivial because its only target is Np63, a node explicitly represented in the keratinocyte differentiation motif.

Step 10: Plugging back the output (TDM) node

Terminal differentiation markers (TDM) are regulated positively through Np63 and negatively through Stat3, the two nodes explicitly represented in the keratinocyte differentiation motif.

Section E: Analytical derivation of necessary conditions for bistability of the keratinocyte differentiation motif

We analysed the $Stat3$ and $Np63$ nullclines of the keratinocyte differentiation motif (*Figure 2A*) to derive the conditions for bistability. The nullclines were obtained by equating $\frac{dStat3(t)}{dt}$ and $\frac{dNp63(t)}{dt}$ (Equation 1) to 0 and solving them individually for Np63.

E.1 Analysis of the Stat3 nullcline

The Stat3 nullcline is obtained by solving $\frac{dStat3(t)}{dt} = 0$ for Stat3 as the Np63-dependent expression, $Stat_{Nullcline}(Np63) = \frac{basal + Calcium + Np63 \cdot v_{Np63}}{d_{Stat3}}$. It is a first-order polynomial of the form of $f(x) = m \cdot x + b$, with slope $m = \frac{v_{Np63}}{d_{Stat3}}$, and an intersection with the ordinate, $b = Stat_{Nullcline}(0) = \frac{(basal + Calcium)}{d_{Stat3}}$, which increases linearly with $basal$ and $Calcium$.

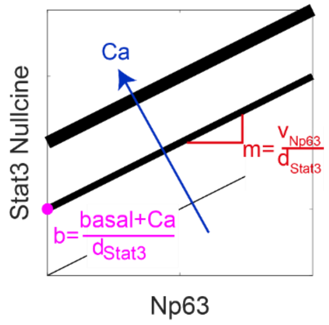

E.2 Analysis of the Np63 nullcline

The $Np63$ nullcline is obtained by solving $\frac{dNp63(t)}{dt} = 0$ for Stat3 as the Np63-dependent expression,

$$Np63_{Nullcline}(Np63) = \frac{Np63 \cdot d_{Np63} - (basal_{Np} + NF\kappa B) - \frac{Np63^{n_H} \cdot va_{Np63}}{Np63^{n_H} + k_{Np63}{}^{n_H}} + Np63 \cdot Calcium \cdot d_{PKC}}{v_{Stat3}}$$

$$= -\overbrace{\frac{(basal_{Np} + NFkB)}{v_{Stat3}}}^{\gamma \in \mathbb{R}^-} + \overbrace{\frac{d_{Np63} + Calcium \cdot d_{PKC}}{v_{Stat3}}}^{\beta \in \mathbb{R}^+} \cdot Np63 - \overbrace{\frac{va_{Np63}}{v_{Stat3}}}^{\alpha \in \mathbb{R}^+} \cdot \frac{Np63^{n_H}}{Np63^{n_H} + k_{Np63}{}^{n_H}}$$

$= \gamma + \beta \cdot Np63(t) - \alpha \cdot \frac{Np63(t)^{n_H}}{Np63(t)^{n_H} + k_{Np63}{}^{n_H}}$. It is in the form of $g(x) = \gamma + \beta \cdot x +$ (Hill function of x) with the lumped parameters, $\gamma = -\frac{(basal_{Np} + NFkB)}{v_{Stat3}}$, $\alpha = \frac{va_{Np63}}{v_{Stat3}}$ and $\beta = \frac{d_{Np63} + Calcium \cdot d_{PKC}}{v_{Stat3}}$.

To understand the geometrical properties of the $Np63_{Nullcline}(Np63)$, let us first investigate the Hill function, - $\alpha \cdot \frac{Np63^{n_H}}{Np63^{n_H} + k_{Np63}{}^{n_H}}$, by assuming $\beta = \gamma = 0$. In this case, the nullcline starts at 0 and monotonically decreases towards $-\alpha$, with the inflection point at $Np63 = k_{Np63}$, with $Np63_{Nullcline}(k_{Np63})$=-$\frac{\alpha}{2}$.

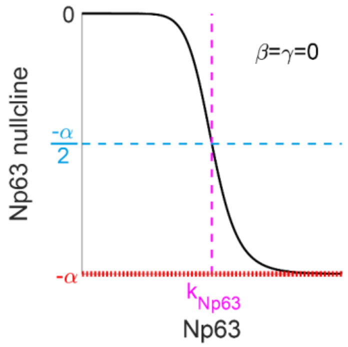

We then add the term $\beta \cdot Np63$ by assuming $\beta > 0$ and $\gamma = 0$, so that $Np63_{Nullcline}(Np63) = \beta \cdot Np63 - \alpha \cdot \frac{Np63^{n_H}}{Np63^{n_H} + k_{Np63}{}^{n_H}}$. The Hill function is rotated around the origin by an angle θ formed by the x-axis and the linear function, $\beta \cdot Np63$. The nullcline converges to

$$\lim_{Np63(t)\to\infty}\left(0 + \beta \cdot Np63 - \alpha \cdot \frac{Np63^{n_H}}{Np63^{n_H} + k_{Np63}{}^{n_H}}\right) = \beta \cdot (Np63) - \alpha.$$

and is bound between $\beta \cdot (Np63) - \alpha$ and $\beta \cdot (Np63)$. The addition of the term $\beta \cdot Np63$ does not change the inflection point at $Np63 = k_{Np63}$ because $Np63_{Nullcline}(k_{Np63}) = \beta \cdot k_{Np63} - \frac{\alpha}{2} = (\beta \cdot k_{Np63} - \alpha) + \alpha/2$.

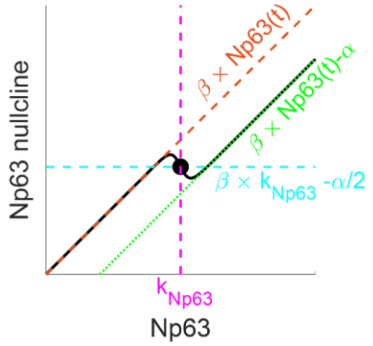

Finally, the entire nullcline $Np63_{Nullcline}(Np63) = \gamma + \beta \cdot Np63(t) - \alpha \cdot \frac{Np63(t)^{n_H}}{Np63(t)^{n_H} + k_{Np63}{}^{n_H}}$ with $\gamma < 0$ shifts the Np63 nullcline along the y-axis.

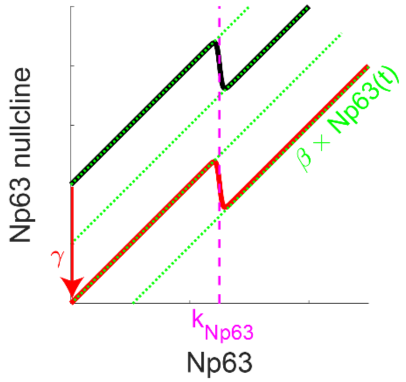

In summary, the Np63 nullcline is an inverted Hill function rotated such that it lies between $(\gamma - \alpha) + \beta \cdot Np63$ and $\gamma + \beta \cdot Np63$. The slope, β, is the only term of the model affected by calcium (PKC).

<u>*E.3 Conditions for the bistability*</u>

The previous sections demonstrated that the $Stat3_{Nullcline}$ is a first order polynomial (Section E.1) and that the Np63 nullcline is given by a rotated Hill function bound between the asymptotes $\gamma + \beta \cdot Np63$ and $(\gamma - \alpha) + \beta \cdot Np63$ (Section E.2). For the system to exhibit bistability, the two nullclines must intersect 3 times, (I) at the asymptotes $\beta \cdot Np63$, (II) at the asymptote $\beta \cdot Np63 - \alpha$ and (III) at the middle segment of the Np63 nullcline.

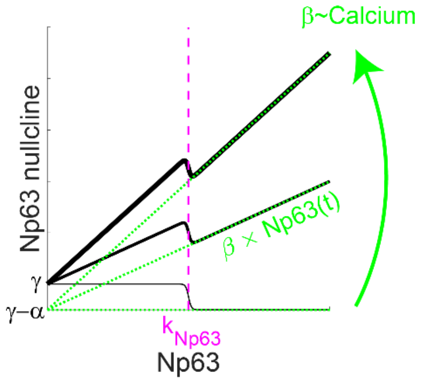

Let the two inflection points of the $Np63_{Nullcline}$ be A* and B*. The conditions for the bistability are

(1) The slope m of the $Stat3_{Nullcline} < \beta$.

(2) The y-intercept of the $Stat3_{Nullcline}$ lies between the y-coordinate of $A^*$and the maximum value between the y-coordinate of $B^*$and 0.

The first condition (1) is described as $m = \frac{v_{Np63}}{d_{Stat3}} < \beta = \frac{d_{Np63} + Calcium \cdot d_{PKC}}{v_{Stat3}}$.

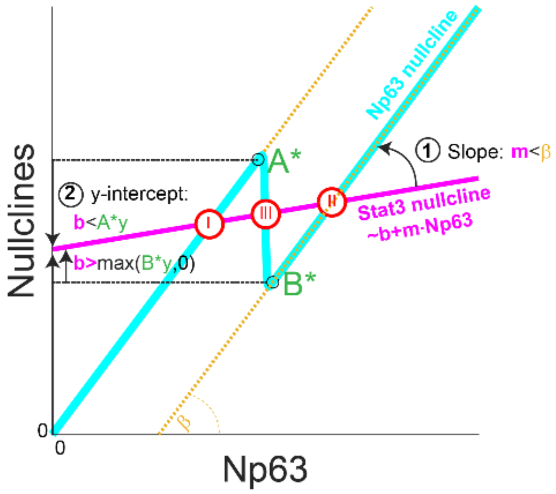

To analytically derive the second condition for bistability, let us assume a large enough Hill coefficient ($nH \rightarrow \infty$) when the Hill function converges to a perfect switch, and derive the coordinates of A* and B*.

When the $Np63$ value is close to k_{Np63}, the Hill function approaches the segment that is orthogonal to $\beta \cdot Np63$ and passes through the point (k_{Np63}, $Np63_{Nullcline}(k_{Np63})$). This segment is described by $Orthogonal(Np63) = -\frac{1}{\beta}Np63 + k_{Np63} \cdot \left(\beta + \frac{1}{\beta}\right) - \frac{\alpha}{2}$, and was obtained by writing an expression for the dot product between $\beta \cdot Np63$ and an unknown vector x, equating this dot product to 0 and finally solving it for x.

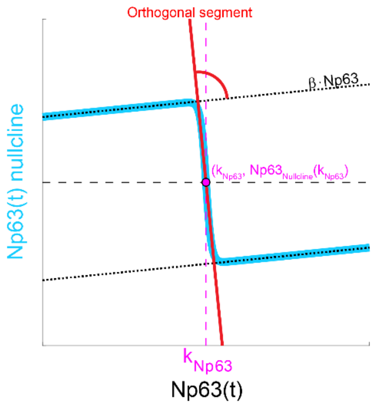

The two inflection points, A* and B*, of the $Np63_{Nullcline}$ as $nH \rightarrow \infty$ are approximated by the intersection points, A and B, of the orthogonal segment with the two asymptotes, $\beta \cdot Np63$ and $\beta \cdot Np63 - \alpha$. Their coordinates are calculated as A$\left(\frac{\mu}{-\beta-\frac{1}{\beta}}, \frac{-\beta\cdot\mu}{-\beta-\frac{1}{\beta}}\right)$ and B$\left(\frac{-\mu-\alpha}{-\beta-\frac{1}{\beta}}, \frac{\frac{\alpha}{\beta}-\beta\cdot\mu}{-\beta-\frac{1}{\beta}}\right)$ with $\mu = k_{Np63} \cdot (\beta + \frac{1}{\beta}) - \frac{\alpha}{2}$. The distance between A and B is $|\overline{AB}| = \sqrt{\frac{\alpha^2}{\beta^2+1}}$. Given that the lumped parameter β is proportional to Calcium ($\beta = \frac{d_{Np63} + Calcium \cdot d_{PKC}}{v_{Stat3}}$, as defined in Section E.2), the larger the input (Calcium) is, the smaller the distance between A and B is.

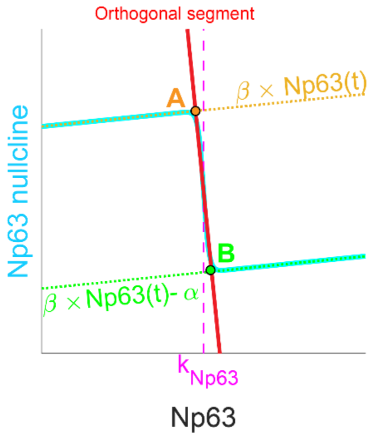

Therefore, the second condition for bistability is given by $b \in \left(\frac{-\beta\cdot\mu}{-\beta-\frac{1}{\beta}}, \max\left(\frac{\frac{\alpha}{\beta}-\beta\cdot\mu}{-\beta-\frac{1}{\beta}}, 0\right)\right)$.

Section F: Model validation

We followed three strategies to validate the model given by Equation 1:

(1) We simulated the reversibility assay in (105) by setting the initial conditions for Equation 1 to the stable steady state value of the Np63 and Stat3 variables corresponding to low calcium condition. For the initial condition of the TDM variable, we set it equal to the corresponding initial experimental timepoint for the TDM. With these initial conditions we numerically integrated for a high calcium pulse for 72h followed by 10 days of a low calcium condition.

(2) We compared the predicted $Np63(t)$ resulting from simulating the calibration dataset with nominal parameters to the measured Np63 levels in Toufighi et al. (77) and in Lena et al. 2008 (46).

(3) We heuristically changed the TDM-specific parameters of Equation 1 to represent the change from primary human keratinocytes used in the calibration dataset to the Human normal epidermal keratinocytes hNEK from which the data in Borowiec et al 2013 (5) were obtained. Calcium levels were

set to PKC=3.3. The initial conditions for Equation 1 correspond to the stable steady state value corresponding to low calcium condition for the Np63 and Stat3 variables, and to the initial experimental timepoint for the TDM variable.

Supplementary Figures

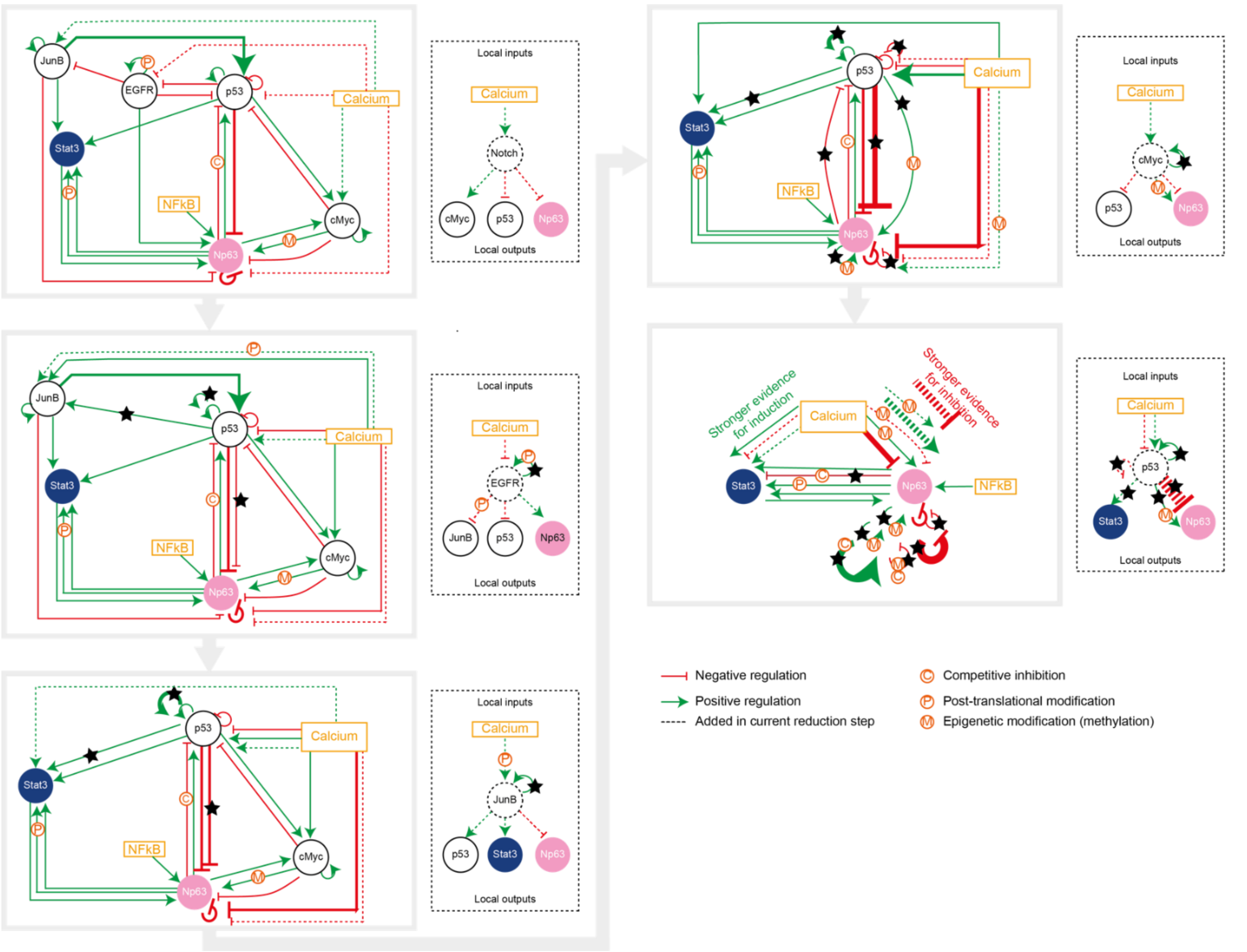

Figure A: *Plugging back inputs (extracellular calcium and infection). Similarly to the network reduction process, we incorporated back the inputs to the reduced keratinocyte differentiation motif by iteratively substituting the Calcium-regulated nodes by its regulatory interactions (through the identification of local inputs and outputs to the nodes).*

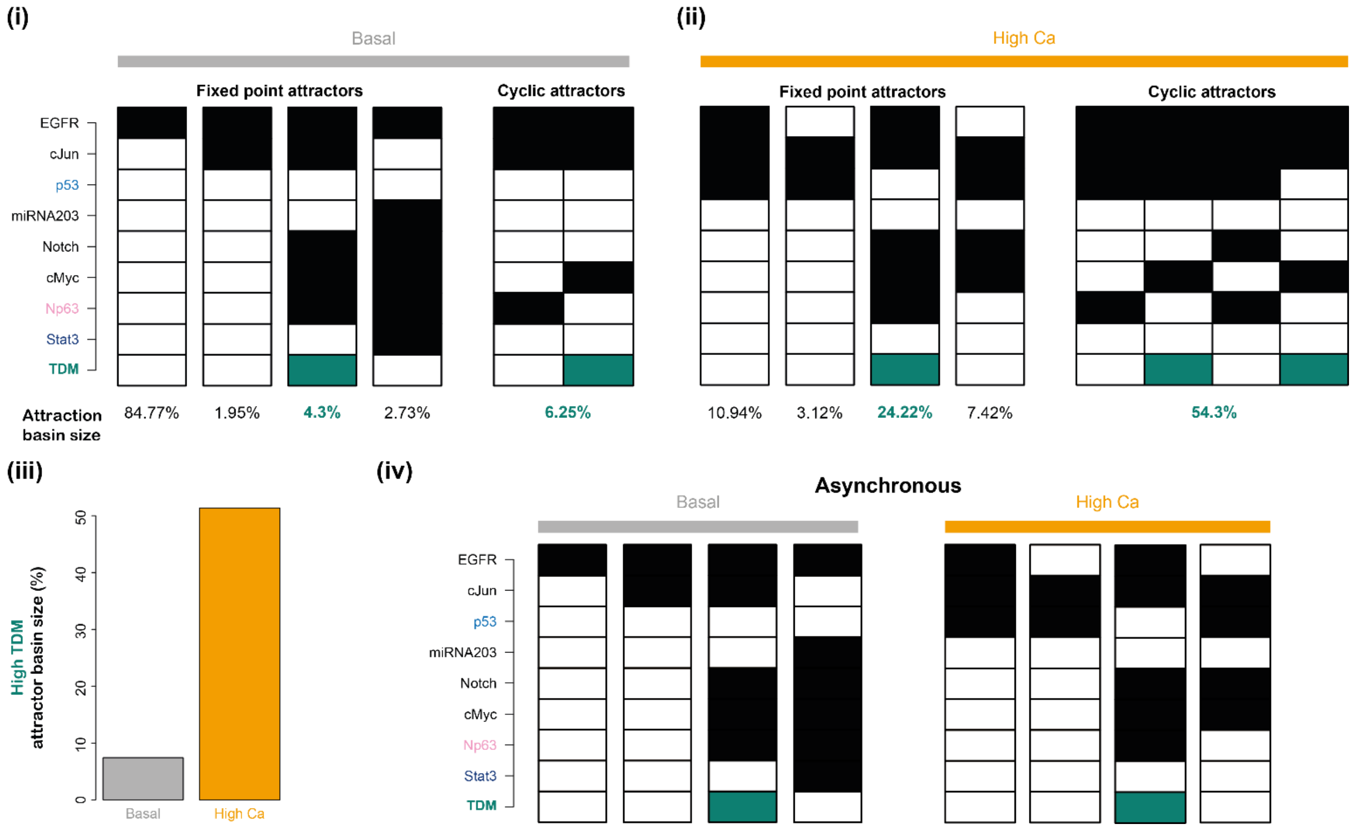

Figure B*: Results of the Boolean model of the regulatory network for keratinocyte differentiation. The model attractors under (i) basal and (ii) high calcium conditions. (iii) The size of the basins of attraction corresponding to the differentiated state under basal and high calcium conditions. (iv) The model attractors under an asynchronous update regime coincide with the fixed-point attractors of the synchronous update regime.*

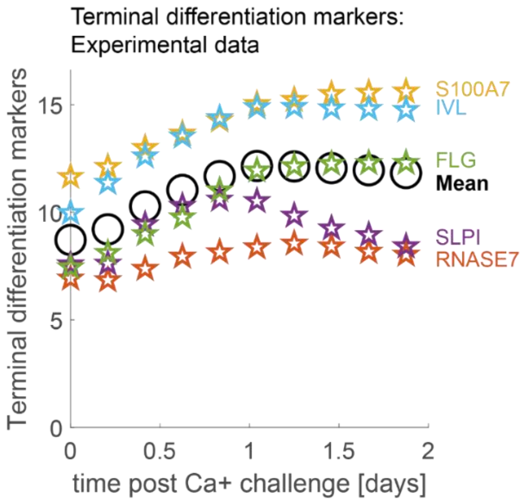

Figure C*: Dynamic, quantitative and high-throughput experimental data from Toufighi et al. (77) shows that the terminal differentiation markers of keratinocyte (the AMP SLPI, S100A7, and RNASE, as well as filaggrin and involucrin) show a slow, steady increase in expressions for 48h upon calcium challenge.*

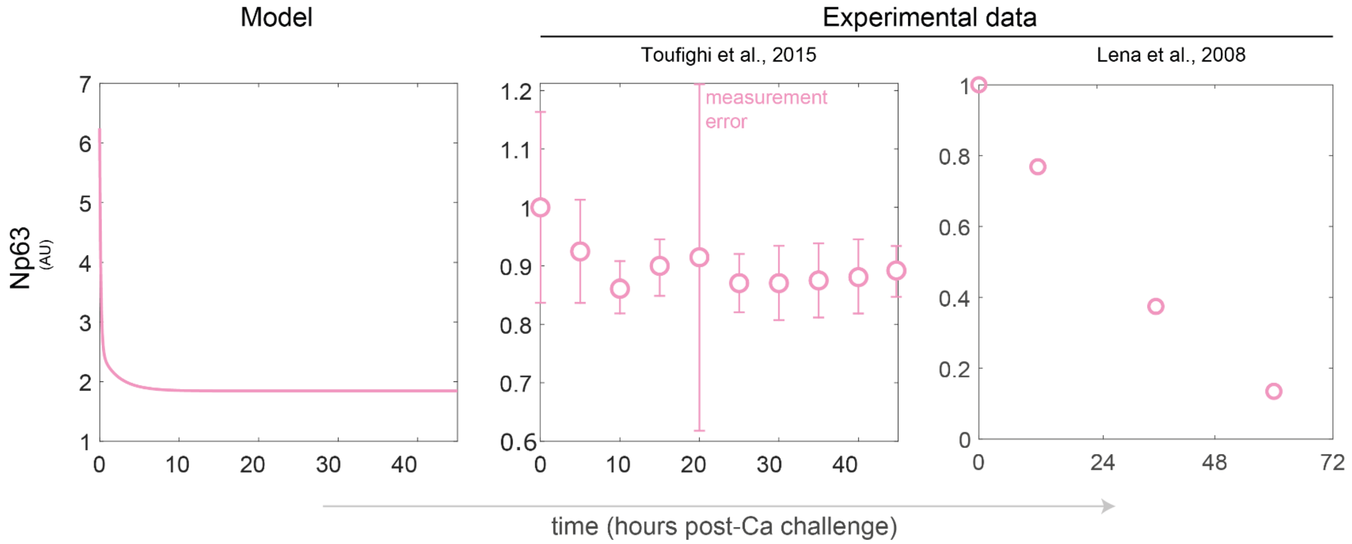

Figure D: *Modelled dynamics of Np63 show a steady decrease in its expression. This dynamical behaviour is qualitatively consistent with the observed reduction in Np63 expression measured in the two independent experiments of Toufighi et al. (77) and Lena et al (46).*

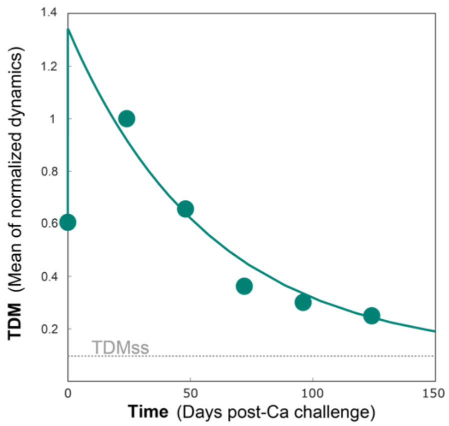

Figure E: *Model reproduces data from Borowiec et al 2013 (5). We heuristically recalibrated the TDM parameters to* a_{TDM}*=1.5,* i_{TDM}*=492;* d_{TDM}*=0.0173;* β*=306 to represent the change from primary human keratinocytes used in the calibration dataset from Toufighi et al.* (77) *to human normal epidermal keratinocytes (hNEK) used in Borowiec et al 2013* (5)*. Calcium levels were set to 3.3.*

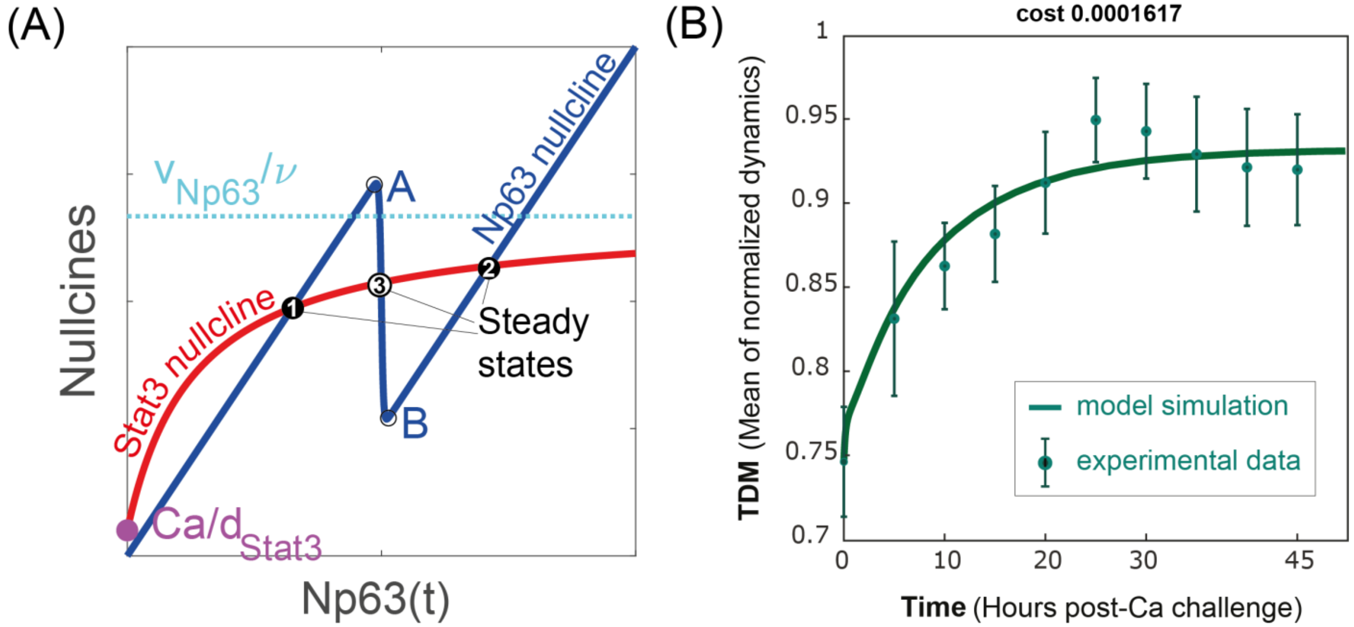

***Figure F:** The keratinocyte differentiation motif is insensitive to adding a negative regulation of Stat3 by Np63 to reflect the incoherent regulation of Stat3 by Np63. (A) Nullcline analysis shows that adding an inhibition of Stat3 by Np63 as a Np63-mediated non-linear degradation term of Stat3 changes the Stat3 nullcline from a linear to a saturated curve that converges to an asymptote that is inversely proportional to the Np63-dependent degradation rate of Stat3. For biologically plausible values of Ca>0, bistability is guaranteed as long as the new asymptote of the Stat3 nullcline passes between the inflection A and B of the Np63 nullcline, as for our original keratinocyte differentiation motif. f(B) The fit to the experimental data is also invariant to adding a Np63-dependent degradation rate of Stat3 to our original model.*

Supplementary References

1. Mascia F, Denning MF, Kopan R, Yuspa SH. The black box illuminated: signals and signaling. Journal of Investigative Dermatology. 2012 Mar;132(3 Pt 2):811–9.
2. Lee SE, Lee SH. Skin barrier and calcium. Ann Dermatol. 2018;30(3):265–75.
3. Celli A, Crumrine D, Meyer JM, Mauro TM. Endoplasmic Reticulum Calcium Regulates Epidermal Barrier Response and Desmosomal Structure. Journal of Investigative Dermatology. 2016;136(9):1840–7.
4. Bikle DD, Xie Z, Tu CL. Calcium regulation of keratinocyte differentiation. Expert Rev Endocrinol Metab. 2013;7(4):461–72.
5. Borowiec AS, Delcourt P, Dewailly E, Bidaux G. Optimal Differentiation of In Vitro Keratinocytes Requires Multifactorial External Control. PLoS One. 2013;8(10):1–15.
6. Saeki Y, Nagashima T, Kimura S, Okada-Hatakeyama M. An ErbB receptor-mediated AP-1 regulatory network is modulated by STAT3 and c-MYC during calcium-dependent keratinocyte differentiation. Exp Dermatol. 2012;21(4):293–8.
7. Koria P, Brazeau D, Kirkwood K, Hayden P, Klausner M, Andreadis ST. Gene expression profile of tissue engineered skin subjected to acute barrier disruption. Journal of Investigative Dermatology. 2003 Aug;121(2):368–82.
8. de Koning HD, van den Bogaard EH, Bergboer JGM, Kamsteeg M, van Vlijmen-Willems IMJJ, Hitomi K, et al. Expression profile of cornified envelope structural proteins and keratinocyte differentiation-regulating proteins during skin barrier repair. Br J Dermatol. 2012 Jun;166(6):1245–54.
9. Gläser R, Harder J, Dressel S, Wittersheim M, Cordes J, Meyer-Hoffert U, et al. Enhanced expression and secretion of antimicrobial peptides in atopic dermatitis and after superficial skin injury. Journal of Investigative Dermatology. 2010;130(5):1355–64.

10. Kisich KO, Carspecken CW, Fiéve S, Boguniewicz M, Leung DYM. Defective killing of Staphylococcus aureus in atopic dermatitis is associated with reduced mobilization of human beta-defensin-3. J Allergy Clin Immunol. 2008 Jul;122(1):62–8.
11. Uribe P, Gonzalez S. Epidermal growth factor receptor (EGFR) and squamous cell carcinoma of the skin: Molecular bases for EGFR-targeted therapy. Pathol Res Pract. 2011;207(6):337–42.
12. Santiskulvong C, Rozengurt E. Protein kinase Calpha mediates feedback inhibition of EGF receptor transactivation induced by Gq-coupled receptor agonists. Cell Signal. 2007;19(6):1348–57.
13. Bheda A, Creek KE, Pirisi L. Loss of p53 induces epidermal growth factor receptor promoter activity in normal human keratinocytes. Oncogene. 2008;27(31):4315–23.
14. Huang L, Pu J, He F, Liao B, Hao B, Hong W, et al. Positive feedback of the amphiregulin-EGFR-ERK pathway mediates PM2.5 from wood smoke-induced MUC5AC expression in epithelial cells. Sci Rep. 2017;7(1):1–12.
15. Kolev V, Mandinova A, Guinea-Viniegra J, Hu B, Lefort K, Lambertini C, et al. EGFR signalling as a negative regulator of Notch1 gene transcription and function in proliferating keratinocytes and cancer. Nat Cell Biol. 2008;10(8):902–11.
16. Yang A, McKeon F. P63 and P73: P53 mimics, menaces and more. Nat Rev Mol Cell Biol. 2000;1(3):199–207.
17. Dotto GP. Crosstalk of Notch with p53 and p63 in cancer growth control. Nat Rev Cancer. 2009;9(8):587–95.
18. Beverly LJ, Felsher DW, Capobianco AJ. Suppression of p53 by Notch in lymphomagenesis: Implications for initiation and regression. Cancer Res. 2005;65(16):7159–68.
19. Mungamuri SK, Yang X, Thor AD, Somasundaram K. Survival signaling by Notch1: Mammalian target of rapamycin (mTOR)-dependent inhibition of p53. Cancer Res. 2006;66(9):4715–24.
20. Nair P, Somasundaram K, Krishna S. Activated Notch1 Inhibits p53-Induced Apoptosis and Sustains Transformation by Human Papillomavirus Type 16 E6 and E7 Oncogenes through a PI3K-PKB/Akt-Dependent Pathway. J Virol. 2003;77(12):7106–12.
21. Boggs K, Henderson B, Reisman D. RBP-Jk binds to and represses transcription of the p53 tumor suppressor gene. Cell Biol Int. 2009;33(3):318–24.
22. Ma X, Li L, Jia T, Chen M, Liu G, Li C, et al. miR-203a controls keratinocyte proliferation and differentiation via targeting the stemness-associated factor D Np63 and establishing a regulatory circuit with SNAI2. Biochem Biophys Res Commun. 2017;491(2):241–9.
23. Lee SH, Lee SJ, Jung YS, Xu Y, Kang HS, Ha NC, et al. Blocking of p53-Snail binding, promoted by oncogenic K-Ras, recovers p53 expression and function. Neoplasia. 2009;11(1):22–31, 6p following 31.
24. Lazzeri E, Peired A, Ballerini L, Lasagni L. Adult Stem Cells in Tissue Homeostasis and Disease. Current Frontiers and Perspectives in Cell Biology. 2012;379–404.
25. Rangarajan A, Talora C, Okuyama R, Nicolas M, Mammucari C, Oh H, et al. Notch signaling is a direct determinant of keratinocyte growth arrest and entry into differentiation. EMBO Journal. 2001;20(13):3427–36.
26. Blanpain C, Lowry WE, Pasolli HA, Fuchs E. Canonical notch signaling functions as a commitment switch in the epidermal lineage. Genes Dev. 2006;20(21):3022–35.
27. Yugawa T, Handa K, Narisawa-Saito M, Ohno S i., Fujita M, Kiyono T. Regulation of Notch1 Gene Expression by p53 in Epithelial Cells. Mol Cell Biol. 2007;27(10):3732–42.
28. Murthy A, Shao YW, Narala SR, Molyneux SD, Zúñiga-Pflücker JC, Khokha R. Notch Activation by the Metalloproteinase ADAM17 Regulates Myeloproliferation and Atopic Barrier Immunity by Suppressing Epithelial Cytokine Synthesis. Immunity. 2012;36(1):105–19.
29. Blanpain C, Lowry WE, Pasolli HA, Fuchs E. Canonical notch signaling functions as a commitment switch in the epidermal lineage. Genes Dev. 2006;20(21):3022–35.
30. Sjöqvist M, Antfolk D, Ferraris S, Rraklli V, Haga C, Antila C, et al. PKCζ regulates Notch receptor routing and activity in a Notch signaling-dependent manner. Cell Res. 2014;24(4):433–50.

31. Raya Á, Kawakami Y, Rodríguez-Esteban C, Ibañes M, Rasskin-Gutman D, Rodríguez-León J, et al. Notch activity acts as a sensor for extracellular calcium during vertebrate left-right determination. Nature. 2004;427(6970):121–8.
32. Sugita S, Hosaka Y, Okada K, Mori D, Yano F, Kobayashi H, et al. Transcription factor Hes1 modulates osteoarthritis development in cooperation with calcium/calmodulin-dependent protein kinase 2. Proceedings of the National Academy of Sciences. 2015;112(10):3080–5.
33. Nickoloff BJ, Qin JZ, Chaturvedi V, Denning MF, Bonish B, Miele L. Jagged-1 mediated activation of notch signaling induces complete maturation of human keratinocytes through NF-κB and PPARγ. Cell Death Differ. 2002;9(8):842–55.
34. Ireland AS, Micinski AM, Kastner DW, Guo B, Wait SJ, Spainhower KB, et al. MYC Drives Temporal Evolution of Small Cell Lung Cancer Subtypes by Reprogramming Neuroendocrine Fate. Cancer Cell. 2020;38(1):60-78.e12.
35. Totaro A, Castellan M, Battilana G, Zanconato F, Azzolin L, Giulitti S, et al. YAP/TAZ link cell mechanics to Notch signalling to control epidermal stem cell fate. Nat Commun. 2017;8(May):1–13.
36. Kopan R, Ilagan MXG. The Canonical Notch Signaling Pathway: Unfolding the Activation Mechanism. Cell. 2009;137(2):216–33.
37. Sasaki Y, Ishida S, Morimoto I, Yamashita T, Kojima T, Kihara C, et al. The p53 family member genes are involved in the Notch signal pathway. Journal of Biological Chemistry. 2002;277(1):719–24.
38. Wu G, Nomoto S, Hoque MO, Dracheva T, Osada M, Lee CCR, et al. DeltaNp63alpha and TAp63alpha regulate transcription of genes with distinct biological functions in cancer and development. Cancer Res. 2003;63(10):2351–7.
39. Yun J, Espinoza I, Pannuti A, Romero D, Martinez L, Caskey M, et al. p53 Modulates Notch Signaling in MCF-7 Breast Cancer Cells by Associating With the Notch Transcriptional Complex Via MAML1. J Cell Physiol. 2015;230(12):3115–27.
40. Boukamp P. Non-melanoma skin cancer: What drives tumor development and progression? Carcinogenesis. 2005;26(10):1657–67.
41. Frye M. Evidence that Myc activation depletes the epidermal stem cell compartment by modulating adhesive interactions with the local microenvironment. Development. 2003;130(12):2793–808.
42. Palomero T, Lim WK, Odom DT, Sulis ML, Real PJ, Margolin A, et al. NOTCH1 directly regulates c-MYC and activates a feed-forward-loop transcriptional network promoting leukemic cell growth. Proceedings of the National Academy of Sciences. 2006;103(48):18261–6.
43. Jensen KB, Watt FM. Single-cell expression profiling of human epidermal stem and transit-amplifying cells: Lrig1 is a regulator of stem cell quiescence. Proceedings of the National Academy of Sciences. 2006;103(32):11958–63.
44. He T. Identification of c-MYC as a Target of the APC Pathway. Science (1979). 1998;281(5382):1509–12.
45. Radoja N, Gazel A, Banno T, Yano S, Blumenberg M. Transcriptional profiling of epidermal differentiation. Physiol Genomics. 2006 Oct 3;27(1):65–78.
46. Yang A, Kaghad M, Caput D, McKeon F. On the shoulders of giants: p63, p73 and the rise of p53. Trends in Genetics. 2002;18(2):90–5.
47. Yi R, Poy MN, Stoffel M, Fuchs E. A skin microRNA promotes differentiation by repressing "stemness." Nature. 2008;452(7184):225–9.
48. Lena AM, Rivetti P, Cervo V, Aberdam D, Knight RA, Melino G, et al. miR-203 represses ' stemness ' by repressing D Np63. Cell Death Differ. 2008;15(1187–1195):1187–95.
49. Melar-New M, Laimins LA. Human Papillomaviruses Modulate Expression of MicroRNA 203 upon Epithelial Differentiation to Control Levels of p63 Proteins ന. J Virol. 2010;84(10):5212–21.
50. Chu WK, Dai PM, Li HL, Chen JK. Transcriptional activity of the ??Np63 promoter is regulated by STAT3. Journal of Biological Chemistry. 2008;283(12):7328–37.

51. Driskell I, Oda H, Blanco S, Nascimento E, Humphreys P, Frye M. The histone methyltransferase Setd8 acts in concert with c-Myc and is required to maintain skin. EMBO Journal. 2012;31(3):616–29.
52. MacPartlin M, Zeng S, Lee H, Stauffer D, Jin Y, Thayer M, et al. P300 Regulates P63 Transcriptional Activity. Journal of Biological Chemistry. 2005;280(34):30604–10.
53. Katoh I, Maehata Y, Moriishi K, Hata RI, Kurata S ichi. C-terminal α Domain of p63 Binds to p300 to Coactivate β-Catenin. Neoplasia (United States) [Internet]. 2019;21(5):494–503. Available from: https://doi.org/10.1016/j.neo.2019.03.010
54. Wang H, Moreau F, Hirota CL, MacNaughton WK. Proteinase-activated receptors induce interleukin-8 expression by intestinal epithelial cells through ERK/RSK90 activation and histone acetylation. The FASEB Journal. 2010;24(6):1971–80.
55. Yalcin-Ozuysal Ö, Fiche M, Guitierrez M, Wagner KU, Raffoul W, Brisken C. Antagonistic roles of Notch and p63 in controlling mammary epithelial cell fates. Cell Death Differ. 2010;17(10):1600–12.
56. Tadeu AMB, Horsley V. Notch signaling represses p63 expression in the developing surface ectoderm. Development. 2013;140(18):3777–86.
57. Nguyen BC, Lefort K, Mandinova A, Antonini D, Devgan V, Gatta G Della, et al. Cross-regulation between Notch and p63 in keratinocyte commitment to differentiation. Genes Dev. 2006;20(8):1028–42.
58. Barbieri CE, Barton CE, Pietenpol JA. ΔNp63α Expression Is Regulated by the Phosphoinositide 3-Kinase Pathway. Journal of Biological Chemistry. 2003;278(51):51408–14.
59. Cheng CC, Wang DY, Kao MH, Chen JK. The growth-promoting effect of KGF on limbal epithelial cells is mediated by upregulation of ΔNp63α through the p38 pathway. J Cell Sci. 2009;122(24):4473–80.
60. Szabowski A, Maas-Szabowski N, Andrecht S, Kolbus A, Schorpp-Kistner M, Fusenig NE, et al. c-Jun and JunB antagonistically control cytokine-regulated mesenchymal-epidermal interaction in skin. Cell. 2000;103(5):745–55.
61. Olsen L, Sherratt J a, Maini PK. A mathematical model for fibro-proliferative wound healing disorders. Bull Math Biol. 1996 Jul;58(4):787–808.
62. Zenz R, Eferl R, Kenner L, Florin L, Hummerich L, Mehic D, et al. Psoriasis-like skin disease and arthritis caused by inducible epidermal deletion of Jun proteins. Nature. 2005;437(7057):369–75.
63. Cavazza A, Miccio A, Romano O, Petiti L, Tagliazucchi GM, Peano C, et al. Dynamic Transcriptional and Epigenetic Regulation of Human Epidermal Keratinocyte Differentiation. Stem Cell Reports [Internet]. 2016;6(4):618–32. Available from: http://dx.doi.org/10.1016/j.stemcr.2016.03.003
64. Angel P, Szabowski A, Schorpp-Kistner M. Function and regulation of AP-1 subunits in skin physiology and pathology. Oncogene. 2001;20(19 REV. ISS. 2):2413–23.
65. Sonkoly E, Wei T, Janson PCJ, Sääf A, Lundeberg L, Tengvall-Linder M, et al. MicroRNAs: Novel Regulators Involved in the Pathogenesis of Psoriasis? PLoS One. 2007;2(7):1–8.
66. Dlugosz AA, Yuspa SH. Coordinate changes in gene expression which Mark the spinous to granular cell transition in epidermis are regulated by protein kinase C. Journal of Cell Biology. 1993;120(1):217–25.
67. Geng Y, Valbracht J, Lotz M. Selective Activation of the Mitogen-activated Protein Kinase Subgroups c-Jun NH 2 Terminal Kinase and p38 by IL-1 and TNF in Human Articular Chondrocytes. Journal of Clinical Investigation. 1996;98(10):2425–30.
68. Muegge K, Vila M, Gusella GL, Musso T, Durum SK. Interleukin 1 induction of the c-jun promoter. PNAS. 1993;90(August):7054–8.
69. Kim BE, Howell MD, Guttman E, Gilleaudeau PM, Cardinale IR, Boguniewicz M, et al. TNF- a Downregulates Filaggrin and Loricrin through c-Jun N-terminal Kinase : Role for TNF- a Antagonists to Improve Skin Barrier. Journal of Investigative Dermatology. 2011;131(6):1272–9.
70. Sonkoly E, Wei T, Pavez Loriè E, Suzuki H, Kato M, Törmä H, et al. Protein kinase C-dependent upregulation of miR-203 induces the differentiation of human keratinocytes. J Invest Dermatol. 2010;130(1):124–34.

71. April T, Mauriello A, Viticchiè G, Lena AM, Latina A, Formosa A, et al. Invasive potential of prostate cancer cell lines MiR-203 controls proliferation , migration and invasive potential of prostate cancer cell lines. Cell Cycle. 2011;10(7):1121–31.
72. Ma X, Li L, Jia T, Chen M, Liu G, Li C, et al. miR-203a controls keratinocyte proliferation and differentiation via targeting the stemness-associated factor D Np63 and establishing a regulatory circuit with SNAI2. Biochem Biophys Res Commun. 2017;491(2):241–9.
73. Lena AM, Cianfarani F, Odorisio T, Melino G, Candi E. MicroRNA-203 contributes to skin re-epithelialization. Cell Death Dis. 2012;3:e435.
74. Jackson SJ, Zhang Z, Feng D, Flagg M, Loughlin EO, Wang D, et al. Rapid and widespread suppression of self-renewal by microRNA-203 during epidermal differentiation. Development. 2013;1891:1882–91.
75. Mckenna DJ, Mcdade SS, Patel D, Mccance DJ. MicroRNA 203 Expression in Keratinocytes Is Dependent on Regulation of p53 Levels by E6. J Virol. 2010;84(20):10644–52.
76. Su X, Chakravarti D, Cho MS, Liu L, Gi YJ, Lin Y li, et al. TAp63 suppresses metastasis through coordinate regulation of Dicer and miRNAs. Nature. 2010;467(7318):986–90.
77. Yi R, Pasolli HA, Landthaler M, Hafner M, Ojo T, Sheridan R, et al. DGCR8-dependent microRNA biogenesis is essential for skin development. PNAS. 2008;106(2):498–502.
78. Wolk K, Witte E, Wallace E, Döcke WD, Kunz S, Asadullah K, et al. IL-22 regulates the expression of genes responsible for antimicrobial defense, cellular differentiation, and mobility in keratinocytes: A potential role in psoriasis. Eur J Immunol. 2006;36(5):1309–23.
79. Zhang L, Li J, Wang Q, Meng G. The relationship between microRNAs and the STAT3-related signaling pathway in cancer. Tumor biology. 2017;1–11.
80. Gao Y, Zhao H, Wang P, Wang J, Zou L. The roles of SOCS3 and STAT3 in bacterial infection and inflammatory diseases. Scand J Immunol. 2018;6(October):1–12.
81. Yi R, Pasolli HA, Landthaler M, Hafner M, Ojo T, Sheridan R, et al. DGCR8-dependent microRNA biogenesis is essential for skin development. Proc Natl Acad Sci U S A. 2009;106(2):498–502.
82. Muhammad N, Bhattacharya S, Steele R, Ray RB. Anti-miR-203 suppresses ER-positive breast cancer growth and stemness by targeting SOCS3. Oncotarget. 2016;7(36):58595–605.
83. Liu H, Zhang Y, Liu Z, Qi H, Zheng X, Qi L, et al. MiR-203 regulates proliferation and apoptosis of ovarian cancer cells by targeting SOCS3. Eur Rev Med Pharmacol Sci. 2019;23:9286–94.
84. Toufighi K, Yang JS, Luis NM, Aznar Benitah S, Lehner B, Serrano L, et al. Dissecting the Calcium-Induced Differentiation of Human Primary Keratinocytes Stem Cells by Integrative and Structural Network Analyses. PLoS Comput Biol. 2015;11(5):1–27.
85. Oh IY, Albea DM, Goodwin ZA, Quiggle AM, Baker BP, Guggisberg AM, et al. Regulation of the Dynamic Chromatin Architecture of the Epidermal Differentiation Complex Is Mediated by a c-Jun / AP-1-Modulated Enhancer. Journal of Investigative Dermatology. 2014;134(9):2371–80.
86. Niehues H, Tsoi LC, van der Krieken DA, Jansen PAM, Oortveld MAW, Rodijk-Olthuis D, et al. Psoriasis-Associated Late Cornified Envelope (LCE) Proteins Have Antibacterial Activity. Journal of Investigative Dermatology. 2017;137(11):2380–8.
87. Meisel JS, Sfyroera G, Bartow-McKenney C, Gimblet C, Bugayev J, Horwinski J, et al. Commensal microbiota modulate gene expression in the skin. Microbiome. 2018;6(1):20.
88. Percoco G, Merle C, Jaouen T, Ramdani Y, Bénard M, Hillion M, et al. Antimicrobial peptides and pro-inflammatory cytokines are differentially regulated across epidermal layers following bacterial stimuli. Exp Dermatol. 2013;22(12):800–6.
89. Sayama K, Komatsuzawa H, Yamasaki K, Shirakata Y, Hanakawa Y, Ouhara K, et al. New mechanisms of skin innate immunity: ASK1-mediated keratinocyte differentiation regulates the expression of β-defensins, LL37, and TLR2. Eur J Immunol. 2005;35(6):1886–95.
90. Calzetti F, Tamassia N, Arruda-silva F, Gasperini S, Cassatella MA. Toluene downregulates filaggrin expression via the extracellular signal-regulated kinase and signal transducer and activator of transcription–dependent pathways. Journal of Allergy and Clinical Immunology. 2017;139(1).

91. Fessing MY, Mardaryev AN, Gdula MR, Sharov AA, Sharova TY, Rapisarda V, et al. p63 regulates Satb1 to control tissue-specific chromatin remodeling during development of the epidermis. J Cell Biol. 2011;194(6):825–39.
92. Romano RA, Ortt K, Birkaya B, Smalley K, Sinha S. An active role of the DN isoform of p63 in regulating basal keratin genes K5 and K14 and directing epidermal cell fate. PLoS One. 2009;4(5).
93. Sen T, Chang X, Sidransky D, Chatterjee A. Regulation of ΔNp63α by NFκB. Cell Cycle. 2010;9(24):4841–7.
94. Katoh I, Maehata Y, Moriishi K, Hata RI, Kurata S ichi. C-terminal α Domain of p63 Binds to p300 to Coactivate β-Catenin. Neoplasia (United States). 2019;21(5):494–503.
95. Wang H, Moreau F, Hirota CL, MacNaughton WK. Proteinase-activated receptors induce interleukin-8 expression by intestinal epithelial cells through ERK/RSK90 activation and histone acetylation. The FASEB Journal. 2010;24(6):1971–80.
96. Borkowski AW, Park K, Uchida Y, Gallo RL. Activation of TLR3 in keratinocytes increases expression of genes involved in formation of the epidermis, lipid accumulation, and epidermal organelles. Journal of Investigative Dermatology. 2013;133(8):2031–40.
97. Duckney P, Wong HK, Serrano J, Yaradou D, Oddos T, Stamatas GN. The role of the skin barrier in modulating the effects of common skin microbial species on the inflammation, differentiation and proliferation status of epidermal keratinocytes. BMC Res Notes. 2013;6:474.
98. Lee SE, Kim JM, Jeong SK, Jeon JE, Yoon HJ, Jeong MK, et al. Protease-activated receptor-2 mediates the expression of inflammatory cytokines, antimicrobial peptides, and matrix metalloproteinases in keratinocytes in response to Propionibacterium acnes. Arch Dermatol Res. 2010 Dec;302(10):745–56.
99. Dommisch H, Chung WO, Rohani MG, Williams D, Rangarajan M, Curtis M a., et al. Protease-activated receptor 2 mediates human beta-defensin 2 and CC chemokine ligand 20 mRNA expression in response to proteases secreted by Porphyromonas gingivalis. Infect Immun. 2007 Sep;75(9):4326–33.
100. Abtin A, Eckhart L, Gläser R, Gmeiner R, Mildner M, Tschachler E. The antimicrobial heterodimer S100A8/S100A9 (Calprotectin) is upregulated by bacterial flagellin in human epidermal keratinocytes. Journal of Investigative Dermatology. 2010;130(10):2423–30.
101. Wanke I, Steffen H, Christ C, Krismer B, Götz F, Peschel A, et al. Skin commensals amplify the innate immune response to pathogens by activation of distinct signaling pathways. Journal of Investigative Dermatology. 2011;131(2):382–90.
102. Müssel C, Hopfensitz M, Kestler HA. BoolNet---an R package for generation, reconstruction and analysis of Boolean networks. Bioinformatics. 2010;26(10):1378–80.
103. Kim JRJ, Kwon YK, Lee HY, Heslop-Harrison P, Cho KH. Reduction of complex signaling networks to a representative kernel. Sci Signal. 2011 Jan;4(175):ra35.
104. Álvarez-Buylla Roces ME, Martínez-García JC, Dávila-Velderrain J, Domínguez-Hüttinger E, Martínez-Sánchez ME. Modeling Methods for Medical Systems Biology [Internet]. Vol. 1069. Springer; 2018. 258 p. Available from: http://link.springer.com/10.1007/978-3-319-89354-9
105. Jadali A, Ghazizadeh S. Protein kinase D is implicated in the reversible commitment to differentiation in primary cultures of mouse keratinocytes. Journal of Biological Chemistry. 2010;285(30):23387–97.
