# Supplementary material for: History-dependent switch-like differentiation of keratinocytes in response to skin barrier damage
Source: PLoS Comput Biol. 2025 Jun 9;21(6):e1013162. doi: 10.1371/journal.pcbi.1013162 (PMC12176289; doi:10.1371/journal.pcbi.1013162)
Supplement: Attachment — Submitted filename: Response to Reviewers.pdf [file pcbi.1013162.s005.pdf]

Reviewer #1

General summary: *In this manuscript, the authors have put together a regulatory network for keratinocyte differentiation and performed Boolean modelling on this network to check if it matches experimental patterns. Then, they performed model reduction to get a minimal motif and performed bifurcation analyses to simulate conditions such as skin barrier damage and infection through Ca and NFkB, respectively. The efforts of the authors in putting together a network that captures keratinocyte differentiation mechanism should be appreciated. However, this study has many major concerns still.*

Response: Thank you for appreciating our efforts and for your constructive feedback.

Comment 1: *It is unclear how many curated publications were used to construct the network: mentioned to be 96 in Line 28 of the abstract, but 85 in Line 120 of the main text, and 85 in Supplementary section S1.*

Response: Thank you for pointing out the inconsistency. We used 101 publications for the construction of the regulatory network. They correspond to the 101 independent citations in Section S1 in the Supplementary material. We have amended the numbers in abstract, main text and Supplementary section S1 accordingly.

Comment 2: *How the inputs and outputs are connected in Figure 1B is not clear, and it would be great if these edges could be added.*

Response: In response to your suggestion, we have added the inputs and outputs in Figure 1B for clarity.

Comment 3: *Additionally, it would be helpful if this network could be provided in a Supplementary table, maybe as an edge list format: source-node, target-node, effect, references.*

Response: Thank you for your suggestion. We have created a new Supplementary Table 1, an edge list format description of the regulatory network.

Comment 4: *What is the reason for the authors to include the boolean simulations in the supplementary section?*

Response: We included the Boolean model analysis in the supplementary material to avoid distracting the readers from our main contribution.

The main contribution of this paper is our proposed *keratinocyte differentiation motif*, which is a minimal, quantitative, dynamic and mechanistic mathematical model of keratinocyte differentiation in response to barrier damage and infection. The construction and analysis of the Boolean model was a necessary intermediate step towards deriving this keratinocyte differentiation motif. It allowed us to validate the consistency between experimental data and the large regulatory network from which we distilled the keratinocyte differentiation motif.

Comment 5: *The presence of cyclic attractors is a bit concerning regarding the interpretation, more so in the High Ca condition. Does this mean that the cell switches back and forth between differentiated and undifferentiated states? Generally, asynchronous boolean tends to have a loss of such cyclic attractors; the authors can perform these simulations and verify if the results of fixed points remain consistent.*

Response: Thank you for pointing this out. Following the reviewer's suggestion, we have performed asynchronous simulations and confirmed that cyclic attractors disappeared while the fixed points remained consistent with synchronous cases.

Accordingly, we have revised the corresponding parts as follows.

- Main text (lines 159-160 of the revised manuscript with Track Changes): We have added a sentence, “We confirmed that the four fixed point attractors are conserved under an asynchronous update regime (Figure S2D).”
- Section S3 (Supplementary material, p. 11): We have added a sentence, “We confirmed that the fixed-point attractors are maintained under an asynchronous update regime” at the end of Section S3.
- Figure S2 (Supplementary material, p. 23): We have added a new panel (D) to show the results of asynchronous simulations.

Comment 6: *I also find the model reduction to be unsatisfactory. For the interaction from Np63 to Stat3, apart from the direct edge and indirect edges via miR-203, the effective regulation through other indirect edges seems to be mostly -ve. Can the authors find the effective regulation between Stat3 and Np63 over the indirect paths and justify the regulations they have considered in the current model?*

Response: Yes, we found the effective regulation between Stat3 and Np63 over the indirect paths. They are described in Steps 1-6 in the network reduction (Section S4). The effects of Np63 on Stat3 are positive through a direct regulation (reference [50] of the Supplementary material) and via miR203, as the reviewer correctly points out.

Comment 7: *In fact, in Section S4, the model reduction gives a higher number of negative interactions from Np63 to Stat3. The authors comment, "For the incoherent regulation (2), we decided to keep only the positive effect of Np63 on Stat3 (Np63 induces Stat3) because adding the negative effect in a mathematical model did not alter the main features of our interest, including observation of the bistable behaviour and the fit to data." This justification seems very unsatisfactory.*

Response: This comment concerns Step 7 (the last step) of the network reduction (Section S4).

The reviewer is correct that the model reduction at Step 7 (when we absorbed the p53 node) gives more negative interactions from Np63 to Stat3. Np63 has 3 negative and 1 positive regulation on p53, which regulates Stat3 through 2 positive interactions. This leads to the 3x2=6 negative regulations and 1x2=2 additional positive regulations from Np63 to Stat3.

Despite these incoherent regulations through p53, we decided to keep only the positive effect of Np63 on Stat3 based on the observation in Response to Comment 6 above and on our further model analysis that we have newly added in a supplementary figure (Figure S6).

The results in Figure S6 demonstrate that adding a negative regulation does not alter the main model behaviour. Specifically, we constructed a mathematical model which includes both a positive and a negative regulation from Np63 to Stat3 and demonstrated that adding a negative regulation from Np63 to Stat3 as an Np63-dependent degradation rate of Stat3 altered neither the bistable behaviour nor the fit to the data. A detailed explanation is provided in the caption of Figure S6.

To clarify the arguments above, we have revised the sentence in question (supplementary material, p. 17) as

> *"For the incoherent regulation (2), we decided to keep only the positive effect of Np63 on Stat3 (Np63 induces Stat3) because the net effect of Np63 on Stat3 has been reported as positive in experimental studies* (50)*. Furthermore, adding the negative effect in a mathematical model did not alter the main features of our interest, including observation of the bistable behaviour and the fit to data (Figure S6)”*

Comment 8: *The validation provided in Figure 2C is also not satisfactory. The peak just after Ca challenge withdrawal seems like an artefact of the model and not a biological trend. However, this cannot be said for sure, given the lack of temporal resolution of data. Regardless, the fit to the rest of the data points is visually bad, and this can be objectively measured by any goodness of fit metric.*

Response: We acknowledge that the model fit is not visually good for model validation in Figure 2C. As the reviewer suggests, most data in the literature has a poor dynamical resolution, and more experiments with a better dynamical resolution are needed to verify whether the transient peak is an artefact of the model.

However, we believe the model captures the qualitative behaviour of the data. Quantitative agreement is not expected between the model simulation and the data used for validation (Figure 2C) because the model was calibrated using the data from primary human keratinocytes, whereas the validation data was from mouse keratinocytes.

We claimed the quantitative agreement of the model with experimental data because we also confirmed that the model simulations reproduced the qualitative trend for 3 additional datasets: the decaying dynamics of Np63 expression observed in two independent experiments (Figure S4) and the dynamics of filaggrin expression observed in a keratinocyte differentiation assay in human epidermal keratinocytes (Figure S5).

To clarify that we reproduced the qualitative (rather than quantitative) behaviour, we have revised the corresponding part (lines 227-228 of the revised manuscript with Track Changes) as

> "We validated our model with calibrated parameters by confirming that the model dynamics reflect the qualitative dynamical behaviour of the reversible keratinocyte differentiation assays".

Comment 9: *The authors should explain their choice of hills functions specifically for the autoinduction of Np63 while using linear terms for the rest of the regulations.*

Response: We used a Hill function to describe the two non-transcriptional events that mediate autoinduction of Np63: The formation of a heteromeric protein complex with p53 (Step 7 of section S4) and methylation via cMyc (Step 6 of Section S4). Formation of a heteromeric protein complex underlies 15 out of the 16 auto-inductions of Np63, meaning that the positive feedback is mediated mainly by this type of non-transcriptional event. In contrast, most other regulations of the keratinocyte differentiation motif involve transcriptional events that are modelled by the law of mass action.

To clarify the choice of Hills function, we have revised the corresponding sentence to the main text (lines 211-214 of the revised manuscript with Track Changes):

> "We describe the auto-induction of Np63 expression by a Hill function (with a maximal rate, va_{Np63}, a half-maximal inductor concentration, k_{Np63}, and a Hill-coefficient, n_H) because it is mediated by the formation of protein complexes."

Comment 10: *Additionally, there seems to be a missing v_{Ca} term for Equation 1a*.

Response: It is not missing. To keep the number of parameters as small as possible, we assume Ca in Equation 1a to be a lumped input variable that represents the effects of calcium on the dynamics of Stat3. It is effectively (calcium concentration)x(its rate of activation of Stat3, *v_{Ca}*) with the unit of (concentration/time) as shown in Table 1.

To clarify this, we added the following explanation (lines 210-211 of the revised manuscript with Track Changes)

> "which is a lumped parameter representing the effects of calcium on the dynamics of Stat3".

Comment 11: *The term (a_{TDM} Np63(t) e^{-\beta t}) does not seem to be the right fit for the process and, consequently, the memory effects described here. The terms seem to be reducing the influence of Np63 and Stat3 on TDM expression over time rather than the methylation of their promoters by Np63. This could also explain why the TDM curve seems to flatten out.*

Response: The symbol * in the term (a_{TDM} Np63(t) * e^{-\beta t}) represents the convolution rather than a simple multiplication. The convolution is used to capture the persistence of the effect of Np63(t) on TDM(t) even after its inactivation. The exponential decay (e^{-\beta t}) represents the system's memory. The faster the decay, the less memory is retained. The flattening out of the TDM curve is not due to the convolution term.

To clarify the above, we have amended the line 217 (of the revised manuscript with Track Changes) as

> "It is modelled with the convolution of $Np63(t)$ with a decaying exponential $(a_{TDM} \cdot Np63(t) * e^{-\beta t})$ with maximal rate, a_{EDC}, and the exponent, β, quantifying the memory of the methylation."

Comment 12: *The authors mention that terminal differentiation is impaired upon increased pathogen load in Line 81. However, their model predicts a decreased threshold for differentiation on increased NFkB, a proxy for pathogen load, and this would imply ease of differentiation. The authors then contradict themselves by mentioning that keratinocyte differentiation is more sensitive in Line 268.*

Response: There seems to be some confusion here. In line 81 (Introduction), we mention that keratinocyte differentiation is "*modulated* by infection, which often *accompanies* skin barrier impairment", rather than "*terminal differentiation is impaired upon increased pathogen load*".

As the reviewer correctly pointed out, we showed that "increasing NFkB in our model lowers the activation thresholds of calcium (C+) required for keratinocyte differentiation" (line 278-279), suggesting that infection has a positive effect on keratinocyte differentiation by increasing the sensitivity to calcium (lines 279-283).

Comment 13: *There were a few places where the references were not linked properly (Line 146, 157).*

Response: Thank you for pointing this out. We have amended the links to the missing references.

Comment 14: *The GitHub link provided is accessible and has a README, but a better organization of the codes could be done along with steps on running the codes.*

Response: Thank you for the suggestion. We have organised the codes by adding comments and expanded the README section to provide a step-by-step explanation of how to run the codes.

Reviewer #2

General comment*: In this study, the authors develop a mechanistic model of keratinocyte differentiation under skin barrier damage and infection by constructing a regulatory network through the systematic curation of experimental study results. Furthermore, they identify a minimal regulatory network, termed the keratinocyte differentiation motif, using the kernel reduction method, and propose a dynamical system of equations to analyze the kinetics of this motif. The model's predictions are subsequently validated using experimental data from in vitro keratinocyte differentiation assays. Their study reveals interesting results such as immune response from infections lowers the threshold Ca levels required for keratinocyte differentiation, etc. The modeling framework and the results presented in this manuscript are convincing. Therefore, I am in favor of the publication of this manuscript in PLOS Computational Biology. Below I am providing a series of comments throughout the manuscript, which I think will improve the manuscript.*

Response: Thank you for your very positive comments and thoughtful suggestions.

Comment 15: *Page 3, Line 77-79: This is a complex sentence conveying information about multiple processes and their causes. It is better to break it into simpler sentences to improve readability.*

Response: We agree with your comment and have revised the sentence to break it into simpler sentences.

Previous version:

> "The skin barrier is maintained by feedback regulation of keratinocyte differentiation triggered by skin barrier damage (2–7) via activation of transcription factors and signalling molecules that induce expression of terminal differentiation markers (8–15)."

Revised version:

> "The skin barrier is maintained by feedback regulation of keratinocyte differentiation. This feedback regulation is triggered by skin barrier damage (2–7) via activation of transcription factors and signalling molecules that induce expression of terminal differentiation markers (8–15)."

Comment 16: *Page3, Line 81: Provide a brief explanation of skin barrier homeostasis here for readers who may not be familiar with this concept.*

Response: Following the reviewer's suggestion, we have added a brief explanation of skin barrier homeostasis.

Revised version:

> "Skin barrier homeostasis is the ability of skin to maintain its barrier function despite external perturbations. Loss of skin barrier homeostasis is associated with impaired terminal differentiation of keratinocytes that results in the formation of a deficient skin barrier, leading to pathological phenotypes such as atopic dermatitis, psoriasis, and cutaneous squamous cell carcinoma (22–24) in which increased pathogen loads prevail."

Comment 17: *Page 3, Line 85: Would it be better to say "impact" rather than "contribute to", given that both factors negatively affect skin barrier homeostasis?*

Response: This comment is about the sentence "Here, we aim to understand how infection and skin barrier damage contribute to skin barrier homeostasis by elucidating how keratinocyte differentiation is regulated by skin barrier damage, especially in the presence of infection."

We respectfully decline the reviewer's suggestion to change "contribute to" to "impact" because our modelling results suggest that both infection and skin barrier damage trigger keratinocyte differentiation, which is necessary to maintain skin barrier homeostasis.

Comment 18: *Page 3, Line 88-90: While this is partially true, there are experimental studies with in vivo animal models and in vitro epidermal or full-thickness skin models (Meesters et al., Keratinocyte signaling in atopic dermatitis: Investigations in organotypic skin models toward clinical application. Journal of Allergy and Clinical Immunology, 151(5), 1231 - 1235.)*

Response: This comment is about the sentence, "Clarifying and predicting the relationship between keratinocyte differentiation, skin barrier damage, and infection is challenging from a purely empirical perspective due to the difficulties in performing quantitative experiments at a cellular level in stratified multi-layered tissue."

We have revised the corresponding sentence and added the reference suggested by the reviewer:

> "Clarifying and predicting the relationship between keratinocyte differentiation, skin barrier damage, and infection is challenging from a purely empirical perspective due to the difficulties in performing quantitative experiments at a cellular level in a stratified multi-layered tissue, despite recent advances in in vitro epidermal or full-thickness skin models (25)."

Comment 19: *Page 4, Line 118: The term "microenvironments" used here is not clearly defined. Does it refer to components such as the extracellular matrix, immune cells, signaling molecules, pathogens, or other factors?*

Response: No, microenvironments used here refer to "the levels and durations of skin barrier damage and infection" defined in lines 106-107.

Comment 20: *Page 4, Figure 1(B): What is PKC? Is it Protein Kinase C? Need an explanation of this abbreviation.*

Response: Yes, PKC is Protein-Kinase C. We have added the explanation of this abbreviation in the caption for Figure 1(B).

Comment 21: *Page 5, Line 141-143: It is nice to have a detailed description of various components of the regulatory network in the Supplementary material. However, it will be better to provide a brief description of the processes and components of the network here. It will help the readers to get an insight into this network and help the flow of the manuscript, without the need to go back and forth between the main text and SM.*

Response: In response to Comment 3 above, we have created a new Supplementary Table 1, an edge list format description of the regulatory network. We hope it will *help the readers to get an insight into this network*. We believe that adding *a brief description of the processes and components of the network* would distract the readers from getting the main story of the paper.

Comment 22: *Page 5, Line 146: Correct the cross-referencing here. There are a few others in the main text and in the SM, please check.*

Response: Thank you for pointing this out. We have amended the links to the missing references.

Comment 23: *Page 5, Line 146-147: Why do you construct a Boolean model, rather than an agent-based model or ODE model? With biologically realistic parametrization, these models provide a realistic quantitative estimation for the dynamical variables. Including a brief explanation of the choice of Boolean model would be helpful.*

Response: We chose a Boolean model to represent the complete regulatory network because this formalism does not require biologically realistic parameters, which are difficult to obtain for systems of ODEs with many coupled variables.

We have added *a brief explanation of the choice of Boolean model* in line 151 (of the revised manuscript with Track Changes):

> "Boolean model allows us to describe the coupled dynamics of the nine state variables without requiring parameter values which are difficult to obtain for large systems."

Comment 24: *Page 6, Line 171-173: How was the decision made regarding which node to remove in the kernel reduction algorithm? Was this choice based on the nature of the available data?*

Response: Yes, this choice was based on the nature of the available data.

To clarify this further, we have revised the explanations in lines 178-181 (of the revised manuscript with Track Changes) as

> "We decided to remove all nodes of the regulatory network except for Stat3 and Np63. We kept Stat3 and Np63 because they directly regulate TDM, and their expression levels are measured in several calcium switch experiments."

Comment 25: *Page 6, Line 184: Is the steady-state line derived from experimental measurements, or does it represent the steady state from the model simulation?*

Response: The steady-state line represents the steady state from the model simulation. To clarify this further, we have amended the corresponding sentence in the Fig 2 (B) caption as

> "The steady state (TDMss) obtained by the model simulation is shown as a grey dotted line. "

Comment 26: *Page 6, Figure 2(B): The y-axis of Figure 2(B) is a bit confusing. What does mean of normalized dynamics mean here?*

Response: We acknowledge that the y-axis label of Figure 2(B) was confusing. We have changed the y-axis label to "TDM (AU)" by deleting "*mean of normalized dynamics"*, which was unclear.

Instead, we have revised the Figure 2B caption to include what we meant by the "mean of normalized dynamics":

> "(B) Model fitting to TDM gene expression in response to a step increase in the calcium concentration (from 0.05 to 1.2 mM CaCl2); data from (8) represents the distribution (mean and standard deviation) over different terminal differentiation marker genes (SLPI, S100A7, and RNASE7, IVL and FLG), each of which was normalized by its maximal value. Error bars represent the dispersion (SD) of the individual genes".

Comment 27: *Page 6, Figure 2(C): It seems to me that in Figure 2(C), the experimental data shows some sort of decay, whereas the model predicts some plateauing. It would be good to provide some statistical tests to make a quantitative assessment of how well the model's predictions match the actual data.*

Response: This comment is related to Comment 8 above.

More experiments with a better dynamical resolution would be needed to verify if late timepoints show a decay or plateauing at an intermediate value. However, we believe the model captures the qualitative behaviour of the data. Quantitative agreement is not expected between the model simulation and the data used for validation (Fig 2C) because the model was calibrated using the data from primary human keratinocytes, whereas the validation data was from mouse keratinocytes. Therefore, performing statistical tests is not relevant here to make a quantitative assessment of how well the model's predictions match the actual data.

We claimed the quantitative agreement of the model with experimental data because we also confirmed that the model simulations reproduced the qualitative trend of 3 additional datasets: the decaying dynamics of Np63 expression observed in two independent experiments (*Figure S4*) and the dynamics of filaggrin expression observed in a keratinocyte differentiation assay in human epidermal keratinocytes (*Figure S5*).

To clarify that we reproduced the qualitative (rather than quantitative) behaviour, we have revised the corresponding part as

> "We validated our model with calibrated parameters by confirming that the model dynamics reflects the qualitative dynamical behaviour of the reversible keratinocyte differentiation assays" in the main manuscript, lines 227-228 of the revised manuscript with Track Changes.

Comment 28: *Page 6, Figure 2C: Similar to the previous comment, would adding more nodes (not reducing the network to just two nodes, but having three or more) improve the model's ability to capture the experimental result.*

Response: Yes, adding more nodes could improve the model's ability to capture the experimental results. However, it would also add more parameters to the model. Given the scarcity of available quantitative and longitudinal data, obtaining good estimates for those parameters would be difficult, increasing the risk of over-

fitting due to increased degrees of freedom by more parameters. There is a risk that a better agreement between experimental data and the simulation results of a larger model could not guarantee a better model but could be attributed to over-parameterization.

To clarify this issue, we have added the following paragraph in the Discussion (lines 311-319 of the revised manuscript with Track Changes).

> "Our proposed keratinocyte differentiation motif comprises the smallest set of variables and their interactions that can reproduce various empirical observations of keratinocyte differentiation in response to calcium and infection derived from different experimental conditions. Adding more nodes could improve the model's ability to capture more experimental results, including the deleterious effects of HPV (42,49,55) and inflammation (54,56,57) on keratinocyte differentiation. However, it would also add more parameters to the model. Given the scarcity of available quantitative and longitudinal data, obtaining reliable estimates for those parameters would be difficult. We hope our work will motivate experimentalists to generate more quantitative time-resolved measurements of keratinocyte differentiation."

Comment 29: *Page 7, Equation (1a): Shouldn't the first term depend on the concentration of calcium as well?*

Response: This comment is related to Comment 10 above.

Yes, the first term of Equation (1a), Ca, depends on the calcium concentration. It is a lumped input variable that represents the effects of calcium on the dynamics of Stat3. It is effectively (calcium concentration)x(its rate of activation of Stat3, *v_{Ca}*) with the unit of (concentration/time) as shown in Table 1.

To clarify this, we added the following explanation (lines 210-211 of the revised manuscript with Track Changes)

> "which is a lumped parameter representing the effects of calcium on the dynamics of Stat3".

Comment 30: *Page 7, Line 202: What does maximal velocity signify here? Is it just the rate?*

Response: Yes, maximal velocity is the maximal rate.

To avoid confusion, we changed the word "velocity" to "rate" in line 213 of the revised manuscript with Track Changes:

> **"**We describe the auto-induction of Np63 expression by a Hill function (with a maximal rate, va_{Np63}, a half-maximal inductor concentration, k_{Np63}, and a Hill-coefficient, n_H) because it is mediated by the formation of protein complexes."

Comment 31: *Page 7, Line 210-211: Provide a brief explanation of how the parameter estimates are obtained using this method.*

Response: The sentence the reviewer is referring to is "We obtained the kinetic model parameters by global optimisation using the dynamic experimental data from primary human keratinocytes".

We have added a brief explanation of how the parameter estimates are obtained in lines 222-224 of the revised manuscript with Track Changes as

> "We obtained the kinetic model parameters by minimising the difference between the model simulation and the dynamic experimental data from primary human keratinocytes (8) using a global optimisation algorithm",

while more detailed explanation is provided in the Methods section.

Reviewer #3

***General summary**: This manuscript presents a mechanistic model of keratinocyte differentiation in response to skin barrier damage and infection. This research provides insights into the mechanisms of skin barrier homeostasis maintenance under fluctuating environmental conditions. The manuscript appears to be technically sound, presenting a well-structured approach to modeling keratinocyte differentiation in response to skin barrier damage and infection. However, the manuscript could be improved if the authors add **more information regarding** several issues.*

Response: Thank you for your very positive comments and thoughtful suggestions.

***Comment 32:** The step-wise approach from Boolean to ODE modeling used in this manuscript has several potential weak points:*

1- *oversimplification in Boolean model, and potential loss of quantitative information: the Boolean model reduces complex interactions to binary on/off states, potentially oversimplifying the system's behavior.*
2- *Similarly, limited temporal resolution: Boolean models typically use synchronous updates, which may not accurately capture the different timescales of biological processes*
3- *challenges in model reduction (potential loss of important interactions)*

Response:

1. We agree with the reviewer that Boolean models cannot capture quantitative information and potentially over-simplify the system's behaviour. That is why a more detailed and quantitative analysis was performed with the ODE model. We used the Boolean model to confirm that the complete regulatory network can capture the calcium-switch experiments because coarse-grained dynamical simulations using the Boolean models are sufficient to confirm the population of differentiated keratinocytes increases dramatically upon increases in calcium (lines 156-165 of the revised manuscript with Track Changes).

2. We agree with the reviewer that synchronous updates may not accurately capture the different timescales of biological processes. We have performed asynchronous simulations and confirmed that the fixed points remained consistent with synchronous cases. The results are shown in the newly added panel (D) of Figure S2.

3. This comment is related to Comment 28 above. We agree with the reviewer that there is a potential loss of important interactions by model reduction (detailed in Section S4). However, we believe that the keratinocyte differentiation motif with two nodes is reasonable, given the scarcity of available quantitative and longitudinal data. Adding more nodes or regulatory interactions to the motif could augment its ability to capture experimental results but with a risk of overfitting. To clarify this issue, we have added the following in the Discussion (lines 311-319 of the revised manuscript with Track Changes).

> "Our proposed keratinocyte differentiation motif comprises the smallest set of variables and their interactions that can reproduce various empirical observations of keratinocyte differentiation in response to calcium and infection derived from different experimental conditions. Adding more nodes could improve the model's ability to capture more experimental results, including the deleterious effects of HPV (42,49,55) and inflammation (54,56,57) on keratinocyte differentiation. However, it would also add more parameters to the model. Given the scarcity of available quantitative and longitudinal data, obtaining reliable estimates for those parameters would be difficult. We hope our work will motivate experimentalists to generate more quantitative time-resolved measurements of keratinocyte differentiation."

Comment 33: *potential limitations of the ODE model and parameter estimation uncertainty: the ODE model requires fitting to experimental data, which can be challenging given the limited availability of quantitative, time-resolved measurements.*

Response: We agree with the reviewer that parameter fitting for the keratinocyte differentiation motif can be *challenging given the limited availability of quantitative, time-resolved measurements.* We have raised this issue and call for the need for more quantitative time-resolved measurements as important future work, in the paragraph added in response to Comment 32-3 above.

Comment 34: *generalizability issues: the models' applicability to different experimental conditions or in vivo situations may be limited, as they are primarily based on and validated against specific in vitro experiments*

Response: We agree with the reviewer that there is a potential issue of generalizability, which is always the case for mathematical models. However, we believe in the proposed models' applicability to different experimental conditions or in vivo situations because our proposed keratinocyte differentiation motif is derived by integrating several sources of experimental data, including 101 experimental observations for the construction of the regulatory network (newly added Supplementary table 1), a dataset for the model parameter estimation (Figure 2B), and 3 datasets for the model validation (Figures 2C, S4 and S5). In addition, we used a dataset to ensure that the results of the Boolean model are consistent with the calcium switch experiments (Figure S2) and 7 datasets with the infection predictions (Figure 4 and lines 281-282).

Comment 35: *Additionally, there are some issues with the writing quality:*

(1) *in the main text, and in suppl material, there are a number of "Error! Reference source not found" instances that should be corrected.*

(2) *there are instances of subject-verb agreement issues and awkward phrasing, such as "the outer skin called skin barrier".*

(3) formatting inconsistencies: references to figures and sections are not consistently formatted throughout the text.

Response: Thank you for this comment.

(1) We have amended the links to the missing references.
(2) We have carefully revised the manuscript and supplementary material to ensure any subject-verb agreement issues and awkward phrasings are avoided.
(3) We have carefully revised the manuscript and supplementary material to ensure that the references to figures and sections are consistently formatted throughout the text.
